# Pediatric SARS-CoV-2 long term outcomes study (PECOS): cross sectional analysis at baseline

**DOI:** 10.1038/s41390-024-03777-1

**Published:** 2024-12-18

**Authors:** Gina A. Montealegre Sanchez, Lauren E. Arrigoni, Alexandra B. Yonts, Kevin B. Rubenstein, James E. Bost, Max T. Wolff, Mallory C. Barrix, W. Patricia Bandettini, Bema Boateng, Dorothy I. Bulas, Thomas R. Burklow, Kayla P. Carlyle, Marcus Chen, Sanchita Das, Robin L. Dewar, Austin A. Dixon, Maureen A. Edu, Rachel L. Falik, Monika L. Geslak, Marcin Gierdalski, Ashraf S. Harahsheh, Linda J. Herbert, Jeroen Highbarger, Saira R. Huq, Arthur Ko, Anastassios C. Koumbourlis, Stephanie R. Lacey, Andrew J. Lipton, Maureen Monaghan, Anta S. Ndour, Laura J. Olivieri, Dinesh K. Pillai, Catherine A. Rehm, Craig A. Sable, Vandana Sachdev, Audrey E. Thurm, Uyen T. Truong, Evrim B. Turkbey, Eric Vilain, Shera Weyers, Jacob S. White, Abigail A. Williams, Jonathan Zember, C. Jason Liang, Meghan Delaney, Mark L. Batshaw, Luigi D. Notarangelo, David L. Wessel, Karyl Barron, Roberta L. DeBiasi

**Affiliations:** 1 Division of Clinical Research (DCR), National Institute of Allergy and Infectious Diseases (NIAID), National Institutes of Health (NIH), Bethesda, MD, USA.; 2 Center for Cancer and Immunology Research (CCIR), Children’s National Research Institute, Washington, DC, USA.; 3 Department of Pediatrics, The George Washington University School of Medicine & Health Sciences, Washington, DC, USA.; 4 Center for Translational Research, Children’s National Research Institute, Washington, DC, USA.; 5 Division of Pediatric Infectious Diseases, Children’s National Hospital, Washington, DC, USA.; 6 Clinical Monitoring Research Program Directorate (CMRPD), National Cancer Institute Frederick National Laboratory for Cancer Research, Frederick, MD, USA.; 7 Division of Biostatistics and Study Methodology, Children’s National Research Institute, Washington, DC, USA.; 8 Clinical Research Directorate (CRD), National Cancer Institute Frederick National Laboratory for Cancer Research, Frederick, MD, USA.; 9 National Heart, Lung, and Blood Institute (NHLBI), NIH, Bethesda, MD, USA.; 10 Department of Diagnostic Imaging and Radiology, Children’s National Hospital, Washington, DC, USA.; 11 Office of Clinical Research Training and Medical Education, Clinical Center (CC), NIH, Bethesda, MD, USA.; 12 Department of Laboratory Medicine (DLM), CC, NIH, Bethesda, MD, USA.; 13 Department of Pathology and Laboratory Medicine, Children’s National Hospital, Washington, DC, USA.; 14 Division of Cardiology, Children’s National Hospital, Washington, DC, USA.; 15 Division of Psychology & Behavioral Health, Children’s National Hospital, Washington, DC, USA.; 16 Division of Intramural Research (DIR), NIAID, NIH, Bethesda, MD, USA.; 17 Center for Genetic Medicine Research, Children’s National Research Institute, Washington, DC, USA.; 18 Division of Pulmonary & Sleep Medicine, Children’s National Hospital, Washington, DC, USA.; 19 UPMC Children’s Hospital of Pittsburgh, Pittsburgh, PA, USA.; 20 National Institute of Mental Health (NIMH), NIH, Bethesda, MD, USA.; 21 Department of Radiology and Imaging Sciences, CC, NIH, Bethesda, MD, USA.; 22 Institute for Clinical and Translational Science, University of California Irvine, Irvine, CA, USA.; 23 Biostatistics Research Branch, NIAID, NIH, Bethesda, MD, USA.; 24 Department of Pathology, The George Washington University School of Medicine & Health Sciences, Washington, DC, USA.; 25 Clinical Research Institute, Children’s National Hospital, Washington, DC, USA.; 26 Laboratory of Clinical Immunology and Microbiology (LCIM), NIAID, NIH, Bethesda, MD, USA.; 27 Department of Critical Care Medicine, Children’s National Hospital, Washington, DC, USA.; 28 Department of Microbiology, Immunology and Tropical Medicine, The George Washington University School of Medicine & Health Sciences, Washington, DC, USA.

## Abstract

**BACKGROUND:**

PECOS is an ongoing study aimed to characterize long-term outcomes following pediatric SARS-CoV-2 infection.

**METHODS:**

This is a cross-sectional analysis of infected and uninfected cohorts at baseline. Participants (0–21 years) with laboratory-confirmed SARS-CoV-2 infection were enrolled as infected. Uninfected were defined as individuals without history or laboratory evidence of SARS-CoV-2 infection. Outcome measures included demographics, medical history, review of symptoms, physical exam, cardiopulmonary evaluation and validated psychological and developmental surveys. Primary outcomes were cohort comparisons for abnormalities on all measures.

**RESULTS:**

654 participants (541 infected, 113 uninfected) completed baseline visits by June 30, 2023. Infected participants were more likely to report constitutional (OR: 2.24), HEENT (OR: 3.74); respiratory (OR: 2.41), or gastrointestinal (OR: 2.58) symptoms. Infected had worse scores in domains of Pain, Fatigue, Global Health, Physical and Cognitive functioning, Mobility and Sleep disturbances when compared to uninfected controls using Patient Reported Outcomes. Cardiopulmonary findings were similar among cohorts.

**CONCLUSIONS:**

The first report of this ongoing longitudinal study demonstrates that infected participants were more likely to report symptoms compared to uninfected controls, which may affect performance and quality of life of these individuals. Longitudinal data will increase understanding of long-term effects of SARS-CoV-2 infection in children. ClinicalTrials.gov Identifier: NCT04830852

## INTRODUCTION

### Background/Rationale

Since the onset of the SARS-CoV-2 pandemic in 2020, over 776,000,000 people of all ages have been diagnosed with COVID-19 and 7 million people have died globally.^1^ While it was initially thought that children were less likely to be infected and experience less severe disease^2^ or sequelae of Coronavirus Disease 2019 (COVID-19) compared to adults,^3^ it is now recognized that children are frequently infected, and are at risk for both short and long-term sequelae,^4,5^ including hospitalizations,^6^ Multisystem Inflammatory Syndrome in Children (MIS-C),^7^ Post Acute Sequelae of COVID (PASC)/Long COVID,^8^ and death.^6^ There continues to be a relative dearth of both active research and published literature regarding the acute and long-term impacts of SARS-CoV-2 infection on children and adolescents compared to adults. Additional research in the pediatric population is necessary, as the old adage that “children are not just small adults” is also true for sequelae of COVID. This was recently demonstrated by the RECOVER study^9^ group in an article illustrating the differences between symptoms of PASC/Long COVID in a cohort of adolescents, school- age children and adults.

While the frequencies of acute and subacute complications of COVID, such as hospitalization, death and MIS-C have dramatically decreased since the first two years of the pandemic^6^ the trajectory of long-term complications, such as PASC/Long COVID, and the burden on society, remains unknown.^10^

PASC is a complex, heterogenous set of persistent symptoms that present in temporal association with a SARS-CoV-2 infection. The World Health Organization (WHO) defines PASC as symptoms which initially occurred within 3 months of a confirmed or suspected SARS-CoV-2 infection, that persist for 2 months or longer, may be ongoing, relapsing or newly presenting, and have an impact on everyday functioning.^8^ Other public health institutions and research groups have defined PASC as the presence of 1 or more symptoms for at least 4 weeks after the acute SARS-CoV-2 infection.^11^ While a single unifying pathologic process has not been identified as the cause of PASC, multiple pathophysiologic mechanisms have been proposed based on studies in PASC patients, recovered COVID patients and uninfected controls. These include viral protein persistence, auto-immunity, aberrant activation of the immune system, endovascular dysfunction and microthrombi, dysautonomia, small fiber neuropathy, neuronal inflammation and microglial activation, dysregulation of the hypothalamus-pituitary-adrenal axis, mitochondrial dysfunction, dysbiosis and reactivation of latent viruses.^12–16^

It is currently estimated that 5–25% of children infected with SARS-CoV-2 go on to develop Long COVID.^17–20^ Long COVID is likely underdiagnosed in children in medically underserved areas, given the relatively greater impact of acute COVID illness in many of those communities.^21^ A study of electronic medical records of 300,000 adults in New York City, with and without COVID-19 infection between March 2020-October 2021 demonstrated that Black and Hispanic individuals had higher frequencies of post-COVID symptoms.^22^ However, the anecdotal experience of health care providers at both pediatric and adult Long COVID clinics has been that the vast majority of patients presenting for Long COVID diagnosis and care are White and Non-Hispanic, suggesting a disparity between those communities with the access and awareness to seek care and those that most need it.^23^ Research and advocacy which can improve community education, identification and management of pediatric PASC patients in underserved areas may help to prevent worsening disparities in health, educational and socioeconomic outcomes.

This study establishes a prospective, longitudinal, diverse cohort of pediatric participants with documented SARS-CoV-2 infection and uninfected controls to identify pediatric-specific long-term sequelae of SARS-CoV-2 infection. Herein, we present a cross sectional analysis of the data at study entry for infected and uninfected cohorts.

## METHODS

### Study design

This multisite, prospective, longitudinal observational study is ongoing at the National Institute of Allergy and Infectious Diseases (NIAID), National Institutes of Health (NIH) in Bethesda, MD, and Children’s National Hospital (CNH) in Washington, DC. The primary objective of the study is to characterize long-term clinical manifestations and potential sequelae following recovery from SARS-CoV-2 infection in children.

The protocol was approved by the CNH Institutional Review Board (IRB) (#Pro00015510). Written Informed Consent was obtained from all participants ≥18 years of age and at least one parent or legal guardian per participant <18 years of age. Written Assent was obtained for all participants 12–17 years of age.

ClinicalTrials.gov Identifier: NCT04830852

### Patient population

#### Eligibility criteria.

Participants (0–21 years) with laboratory-confirmed history of symptomatic or asymptomatic SARS-CoV-2 infection were enrolled at least 4 weeks from the onset of their acute illness or positive test as infected participants, whichever occurred first. Laboratory confirmation included a positive SARS-CoV-2 reverse transcriptase polymerase chain reaction (RT-PCR), antigen, or anti-nucleocapsid antibody.

Uninfected participants including household contacts, were defined as individuals (0–21 years) without past medical or current history of SARS-CoV-2 infection, in conjunction with negative SARS-CoV-2 RT-PCR and anti-nucleocapsid antibody testing at baseline visit. Individuals enrolled as uninfected but found to have evidence of current or recent SARS-CoV-2 infection at the time of their first protocol visit were discontinued as uninfected and invited to enroll as infected participants.

Participants were primarily recruited from inpatient and outpatient units at Children’s National Hospital (CNH) and CNH Integrated Network, as well as regionally using IRB approved advertisements at the NIH Clinical Center (CC) and in the community. Participants could self-refer and were financially compensated for their time.

#### Baseline evaluation.

All participants underwent a demographic survey, comprehensive medical history including COVID-19 vaccination status, WHO case report form (CRF) for symptoms,^24^ and detailed physical exam. Infected participants provided details of their first and any subsequent COVID-19 infections. Nasopharyngeal (NP), blood, urine, and stool samples were collected and stored in the biorepository.

All participants completed a comprehensive cardiopulmonary evaluation, including chest computed tomography (CT) or chest radiograph (CXR), electrocardiogram (ECG), echocardiogram, 6 min Walk Test (6MWT), and age-appropriate pulmonary function testing (PFT). Cardiac magnetic resonance imaging (MRI) was obtained in a subset of participants and will be summarized in a future manuscript. Legal guardians and participants (dependent on age) completed Developmental Profile-4 (DP4),^11^ selected domains of the Patient Reported Outcomes Measures Information System (PROMIS),^25^ and the CoRonavIruS health Impact Survey (CRISIS)^26^ to assess global functioning, as well as social and health impacts of COVID-19 on quality of life. All questionnaires were completed within Research Electronic Data Capture system (REDCap).^27^

Additional information can be found in the Online material (Supplementary eMethods, Supplementary eFig. 1, Supplementary eTables 1 and 2).

### Statistical analysis

Cohort comparisons were made using generalized estimating equations (GEE). Each family was considered a cluster, and correlation structure was assumed to be exchangeable. For continuous outcomes, an identity link was used; for binary outcomes, a logit link was used. Variable adjustment was done by including age and sex in the GEE model as additive terms. For comparisons of binary outcomes where one group had zero outcomes, the GEE model did not converge; to address this issue, we chose the largest cluster in the group and set the outcome of the cluster’s median aged individual to one. By slightly biasing the comparison towards the null, this allowed the model to converge, and these are the results reported. Additionally, we performed a complementary analysis using Fisher’s exact test where clustering was ignored and therefore the results would be expected to be biased away from the null.

All *p*-values are two-sided. Adjustment variables were specified prior to analysis based on a subjective synthesis of literature review and clinical experience. Missing data was minimal, the extent of which was reported in table and figure legends, and when present, assumed to be missing completely at random.

All analyses were performed using R software, version 4.3.0 (R Foundation for Statistical Computing) or Stata, version 18.0 for windows.

The study was initially designed to recruit a minimum of 710 infected and 355 uninfected participants. However, given the ongoing exposure to SARS-CoV-2 infection in the general population, the recruitment rate for uninfected participants was lower than expected.

Post hoc analyses were completed to assess differences when comparing 6-minute walk test distance adjusting by age, and laboratory abnormalities stratified by time of infection.

## RESULTS

### Study population/demographics

#### Enrollment.

For the period between July 19, 2021, to June 30, 2023, 8236 potential participants were contacted via email, flyer, or telephone calls. Of these, 1765 were screened; 82% (1445/1765) were found to be eligible, and 55% (978/1765) enrolled, of which 720 (74%) were infected and 258 (26%) were uninfected participants. The number of infected participants completing the baseline visit was 559/720, of which 541/559 were categorized as acute COVID-19 and 18/559 were MIS-C cases. Data summarized in this publication include 654 enrolled participants (541 infected and 113 uninfected) who had completed baseline visits between July 19, 2021, and June 30, 2023. MIS-C cases are described separately and not included in subsequent analysis due to the small number. Follow-up protocol study visits are ongoing.

**Fig. 1:**
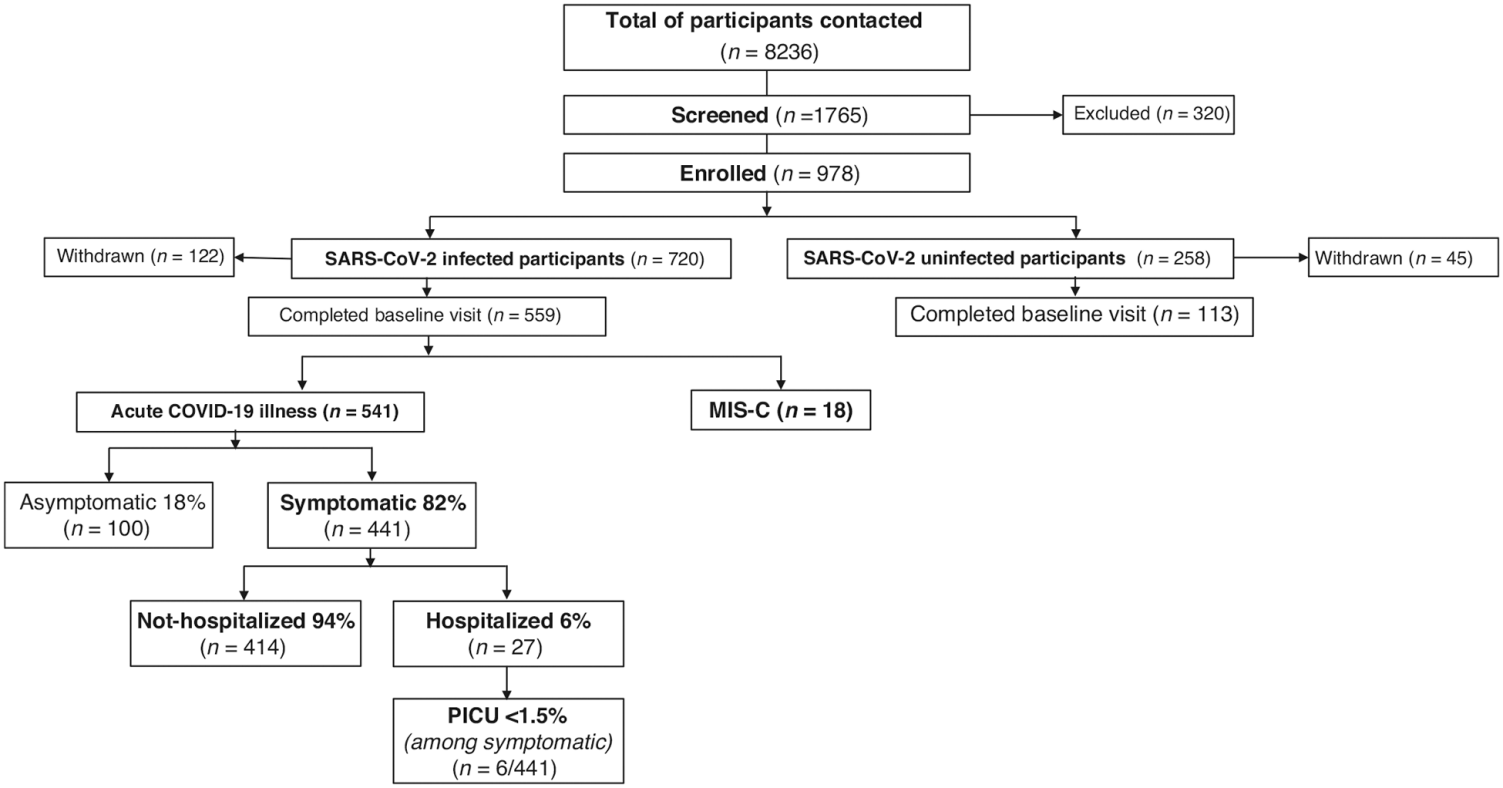
Consort diagram - enrollment summary through June 30, 2023. Flow chart of overall enrollment from July 19, 2021, to June 30, 2023, and flow chart distribution of acute COVID-19 illness and MIS-C cases among infected participants. Baseline visits completed after July 1, 2023, are not included in this report. COVID-19 Coronavirus Disease 2019, MIS-C Multisystem Inflammatory Syndrome in Children, PICU Pediatric Intensive Care Unit.

Among infected participants, 82% were classified as symptomatic; 18% asymptomatic based on the presence or absence of SARS-CoV-2 symptoms at the time of infection. The majority (94%) of symptomatic participants were not hospitalized; only 6% had moderate or severe disease requiring hospitalization, and <1.5% required admission to the Intensive Care Unit (Fig. 1). 33 participants classified as uninfected at enrollment were confirmed to have had SARS-CoV-2 infection at or before the baseline visit and were re-enrolled as infected participants.

#### Demographics.

Overall participant demographics were similar comparing infected and uninfected participants (Table 1). The mean age of the overall cohort was 9 years, with broad and similar representation across all age bands in both cohorts. Overall, 47% of the participants were female, 56% White (higher in uninfected; *p* = 0.03), and 25% Black (higher in infected; *p* = 0.02). 14% of participants identified as Hispanic ethnicity.

#### Underlying medical conditions prior to 2020.

Pre-existing medical conditions were present at baseline in 59% of all participants and were similar in both cohorts (Table 1). Asthma was the most reported medical diagnosis present prior to the date of onset of SARS-CoV-2 circulation, reported by 15% of all participants; followed by neurodevelopmental, and psychiatric diagnoses present in more than 10% of participants (Supplementary eTable 3).

#### SARS-CoV-2 infection timing from initial infection to baseline visit.

The median time between onset of COVID-19 symptoms and baseline visit was 7.3 months (interquartile 4.1–12.3 months) (Table 1). Recruited infected participants were infected during periods in which Wild-type, Alpha, Delta, and Omicron variants were circulating in the US (Supplementary eFig. 2).

#### SARS-CoV-2 vaccination, breakthrough infections, and reinfection.

83% (542/654) of participants were eligible for COVID-19 vaccination at the time of their baseline visit. Of those eligible, 41% (223/542) had received 3 or more doses, with the majority of these being uninfected participants (*p* = 0.02); 40% (215/542) had received 2 doses, 3% (17/542) had received one dose, and 15% (83/542) were unvaccinated. Most eligible unvaccinated participants were infected participants (*p* = 0.01) (Table 1).

Breakthrough infection was defined when SARS-CoV-2 infection occurred at least 14 days after completion of the COVID-19 vaccine regimen. Vaccine regimen completion was defined as either the date of the first Johnson & Johnson vaccine, or the second vaccine of another type. 149 (54%) of first infections in the 278 infected participants who met the criteria for completion of vaccine regimen were considered breakthrough infections.

Re-infection with SARS-CoV-2 occurred in 11% of infected participants by the time of the baseline visit, with 92% of these reinfections classified as mild infection.^28^

#### Differences in infected participant demographics by race and ethnicity.

Differences among infected participants were evaluated by race and ethnicity (non-Hispanics White vs. minority). Descriptive analysis showed similar distribution in the frequency of pre-existing medical conditions prior to 2020 (60% vs. 58%; respectively), as well as the number of participants eligible for COVID vaccine (83% vs. 82%). The percentage of unvaccinated participants who were eligible for vaccination was significantly increased in the minority group (23% vs 9%; *p* = 0.001).

#### MIS-C.

Cases were limited to 18 participants, with a median age of 9.5 years, predominantly non-Hispanic White males. All MIS-C participants were hospitalized with 72% requiring Intensive Care Unit admission. 11/18 (61%) were eligible for COVID-19 vaccination at the time of their baseline visit with 73% receiving 2 or more vaccine doses and 27% remaining unvaccinated. Due to the small number of MIS-C cases enrolled, these subjects were not included in further detailed analysis.

### Baseline visit assessments

#### Reported symptoms at baseline.

Infected were more likely than uninfected participants to report constitutional (OR: 2.24; 95% CI: 1.36–3.67, *p* = 0.001), head, eyes, ears, nose and throat (HEENT) (OR: 3.74; 95% CI: 1.12–12.48, *p* = 0.03), respiratory (OR: 2.41; 95% CI: 1.19–4.88, *p* = 0.01), or gastrointestinal (OR: 2.58; 95% CI: 1.64–4.07, *p* < 0.001) symptom clusters at baseline (Supplementary eFig. 3). These results were not significantly altered when the model was adjusted for pre-existing medical conditions.

**Fig. 2:**
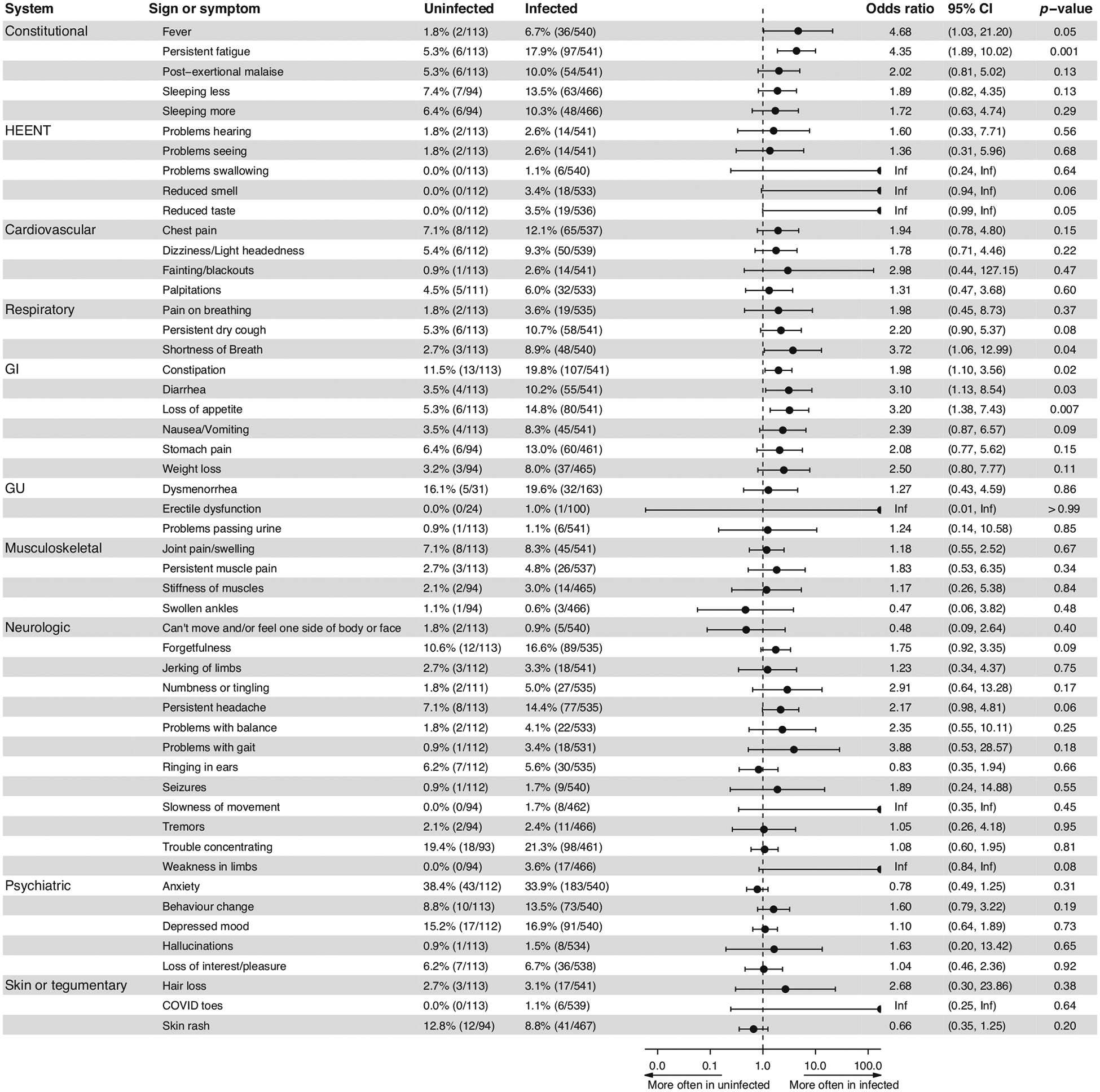
Comparison of review of systems by symptoms in infected and uninfected. Percentage of participants reporting symptoms since recovery of SARS-CoV-2 infection (infected) or since 2020 (uninfected), with odds ratio reporting the odds of infected participants reporting symptoms comparing to uninfected. *P*-values comparing odds of the findings between infected and uninfected are adjusted for age and sex. Generalized estimating equations (GEE) are used to account for correlation between family members participating in the study. CI Confidence Interval, HEENT head, ear, nose, and throat, GI gastrointestinal, GU genitourinary, Inf infinitive.

Analysis by symptoms was consistent with infected participants more commonly reporting persistent fever (OR: 4.68; 95% CI: 1.03–21.20, *p* = 0.05), persistent fatigue (OR: 4.35; 95% CI: 1.89–10.02, *p* = 0.001), shortness of breath (OR: 3.72; 95% CI: 1.06–12.99, *p* = 0.04), constipation (OR: 1.98; 95% CI: 1.10–3.56, *p* = 0.02), diarrhea (OR: 3.10; 95% CI: 1.13–8.54, *p* = 0.03), or loss of appetite (OR: 3.20; 95% CI: 1.38–7.43, *p* = 0.007). The following symptoms were present exclusively in infected participants: weakness in limbs (3.6%), reduced smell (3.4%) or taste (3.5%), slowness of movement (1.7%), problems swallowing and COVID toes (1.1%) (Fig. 2).

#### Physical exam at baseline.

There were no significant differences between cohorts with respect to abnormalities on physical exam. The most common abnormalities detected in both cohorts were enlarged lymph nodes and skin findings, with similar incidence in both (Supplementary eFig. 4).

#### New diagnoses since 2020.

**Fig. 3:**
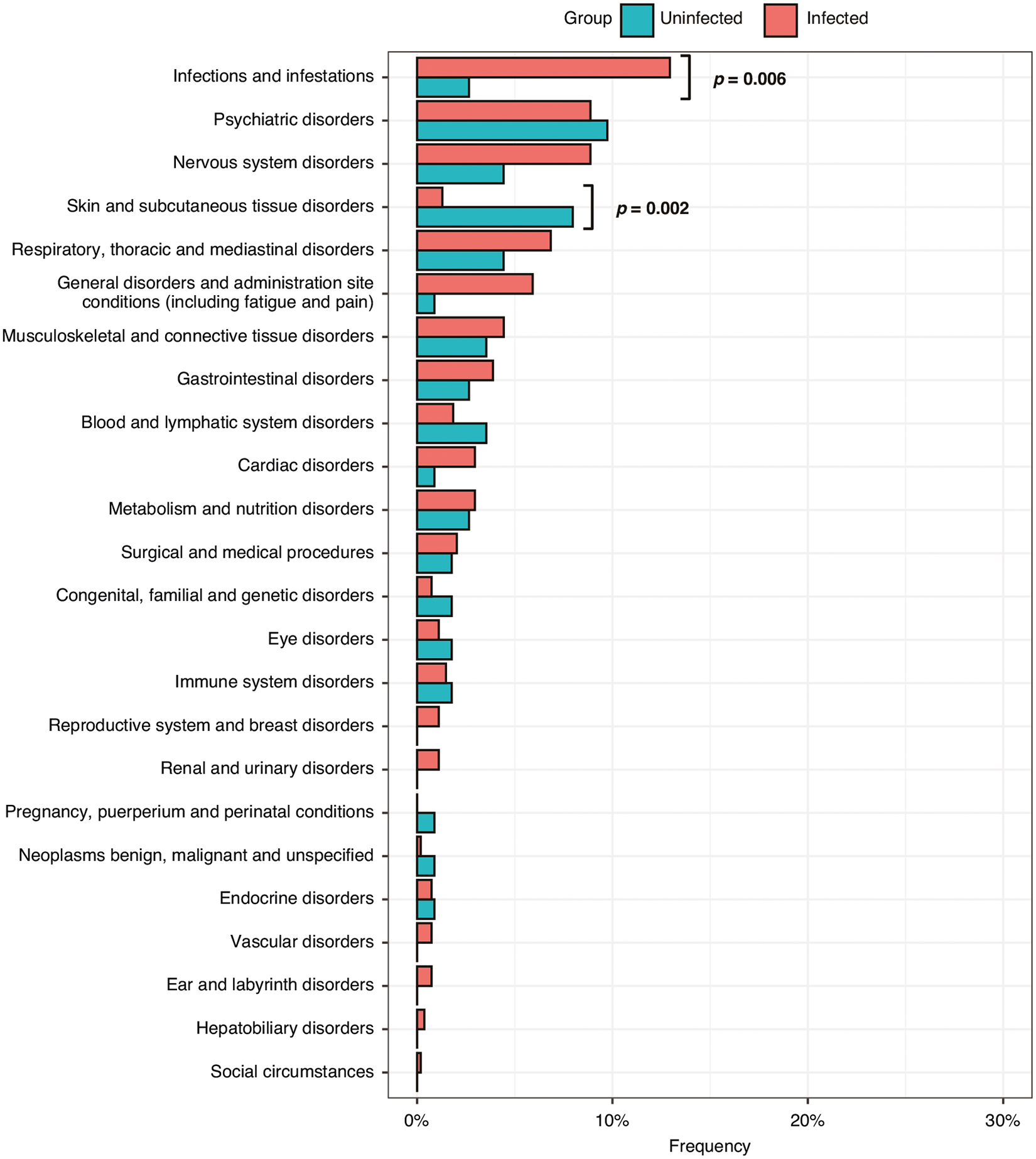
Frequency of new diagnosis in infected (Post-COVID) and uninfected (After January 1, 2020). Medical Dictionary for Regulatory Activities (MedDRA) coding was used for standardization and organ system grouping. SARS-CoV-2 re-infections were excluded from the infections and infestations organ group. MedDRA Categories related to injuries, and investigations are not shown here.

Both infected and uninfected participants received new medical diagnoses since January 2020, and these were grouped by organ system using Medical Dictionary for Regulatory Activities (MedDRA) coding. Within this classification, infectious, neurological, respiratory, musculoskeletal, gastrointestinal, and general disorders were more frequently reported by infected versus uninfected participants (Fig. 3). Viral infections following SARS-CoV-2 infection were more frequently reported by infected participants vs. uninfected (*p* = 0.006).

#### Radiological imaging (CXR and chest CT).

Overall, there was a low incidence of radiographic abnormalities detected at baseline in both cohorts. Infected participants were more likely than uninfected to have CXR abnormalities (OR 3.15; 95% CI 1.34–7.42; *p* = 0.009), primarily increased incidence of perihilar peribronchial thickening (20% vs. 10%, *p* = 0.04), more common in younger infected participants (<4 years) and often associated with a history of respiratory infections, or asthma (Supplementary eFig. 5, Supplementary eTable 4). In contrast, chest CT findings, including ground glass opacities (GGO), reticular/linear opacities, and bronchial wall thickening were similar in both groups. Nodules were the most common finding in both cohorts (30% overall), and were clinically insignificant^29^ in size (<4 mm).

#### Pulmonary function testing and assessment of aerobic capacity and endurance.

There were no significant abnormalities or differences detected between cohorts on baseline lung volumes and adjusted diffusion capacity of the lungs for carbon monoxide (DLCO). Spirometry revealed non-clinically significant increased Forced Expiratory Volume in 1 s/ Forced Vital Capacity (%) in infected vs. uninfected (98 vs. 96, *p* = 0.04). The 6MWT was consistent with a shorter mean distance walked by older infected vs. uninfected participants in the older (>16 years) subgroup. (466.20 vs. 534.56 meters, *p* = 0.02) (Supplementary eTables 5–8).

#### Cardiology evaluation.

There were no significant differences between cohorts in baseline ECG abnormalities, but findings of abnormal T waves, ST and atrioventricular block were only seen in infected participants. There was a statistically non-significant trend for abnormal echocardiogram results in infected compared to uninfected participants, including an increased incidence of mild myocardial systolic dysfunction (shortening fraction <28%) (*n* = 6), small pericardial effusion (*n* = 3), and mild mitral valve regurgitation (*n* = 4). None of these abnormalities were present in uninfected except for 1 participant with mild myocardial systolic dysfunction. No differences in mean coronary artery Z scores were detected among cohorts, but 4 males with mild COVID illness diagnosed between December 2021 to May 2022 had detectable coronary artery dilation (*n* = 3), and one had a small coronary aneurysm, documented within 6 months of their initial SARS-CoV-2 infection (Supplementary eTables 9–11). Follow-up echocardiograms available in 2 of these infected participants were normal at the 12-month visit.

### Psychological and quality of life instruments

#### PROMIS.

Parent and child-reported performance scores (median T scores) were significantly worse in 2 of 10 domains in infected compared to uninfected participants. Specifically, these domains were Pain Interference (*p* = 0.001, *p* = 0.008; respectively) and Fatigue (*p* < 0.001, *p* = 0.01; respectively). In addition, parents of infected participants reported worse scores in the domains of Global Health (*p* = 0.004), Physical Functioning and Mobility (*p* = 0.005), Cognitive Functioning (*p* = 0.006), and Sleep Disturbance (*p* = 0.02) compared to parents of uninfected participants (Table 2).

The proportion of infected participants identified as having a clinically elevated score (≥1 Standard Deviation (SD) worse T score) was significantly greater than in the uninfected group for the following domains: Global Health (18.3% vs. 9.6%, *p* = 0.02), Physical Functioning and Mobility (11.5% vs. 2.3%; *p* = 0.02), Pain Interference (20% vs. 5.8%, *p* = 0.006), Sleep Disturbance (33.5% vs. 21.2%; *p* = 0.02). and Fatigue (19.2% vs. 4.7%, *p* = 0.003) (Supplementary eTable 12).

There were no differences between cohorts for median T scores within the domains of Anxiety, Depressive Symptoms, or Peer Relationships (Table 2). However, there was a substantial and similar proportion of participants in both cohorts identified to have a clinically elevated score in these domains (Supplementary eTable 12 and Supplementary eFig. 6).

#### CRISIS.

Baseline *CRISIS* surveys identified that uninfected participants were more worried about COVID infection than infected. (*p* = 0.02) (Supplementary eTable 13).

### Laboratory analysis

The cohort underwent a comprehensive set of laboratory testing at baseline. A higher percentage of uninfected participants had leukocytosis (*p* = 0.03), including higher absolute neutrophils (*p* = 0.03) and eosinophils counts (*p* = 0.02) (Supplementary eTable 14). There were no differences comparing infected and uninfected regarding percentage of participants with results outside the normal range for other tests studied. A sub-analysis revealed that C4 levels below the normal limits were more frequently observed in infected participants seen within 180 days from their primary infection compared to greater than 180 days (*p* = 0.005). In contrast, aminotransferase, total bilirubin, and absolute lymphocyte count above the normal limits were more frequently observed in infected participants evaluated beyond 180 days of their primary infection (*p* = 0.05; *p* = 0.009; *p* = 0.02 respectively) (Supplementary eTable 15). We hypothesized that a 180-day cut-off would differentiate between early vs. late changes.

#### SARS-CoV-2 PCR and antibodies.

All participants underwent testing at baseline for presence of SARS-CoV-2 virus by PCR, SARS-CoV-2 anti-spike neutralizing antibodies, and anti-nucleocapsid antibodies. Very few participants had detectable viral load at the time of baseline visit (1.7% infected; 0% uninfected), as expected. The lack of detectable anti-nucleocapsid antibodies in uninfected participants confirmed their status. In contrast, the majority (80.5%) of infected participants had detectable anti-nucleocapsid antibodies (*p* < 0.001). A significantly larger percentage of infected compared to uninfected participants had detectable total neutralizing antibody (87.9% vs. 78.2%; *p* = 0.002) (Supplementary eTable 16).

#### Association of neutralizing antibody with vaccination.

Additional analysis was performed to assess the quantitative level of detectable total neutralizing antibodies in infected and uninfected subjects, and the association with number of vaccines received. Infected participants receiving 1–3+ doses of vaccine had higher measurable levels of neutralizing antibody present than unvaccinated infected subjects (96% vs 50% signal inhibition, *p* < 0.001), noting that even infected participants who had not yet received any vaccinations had elevated levels of neutralizing antibody present compared to unvaccinated uninfected subjects (50% vs 0% signal inhibition, *p* < 0.001). In contrast, uninfected participants who had 0–1 vaccine doses had no measurable levels of neutralizing antibodies present and did not reach higher detectable levels until they had received 2–3+ doses of vaccine (1% vs 96% signal inhibition, *p* < 0.001) (Supplementary eFig. 7).

## DISCUSSION

We describe the differences at baseline study entry between pediatric SARS-CoV-2 infected and uninfected participants enrolled in a prospective longitudinal observational study.

In this initial analysis, we offer a wide representation of a group of children and adolescents across age, sex, race, and ethnicity such that findings may be generalizable to the pediatric population who have been affected by SARS CoV-2 infection. Reflective of the pediatric experience during the pandemic, it is important to note that the majority of children enrolled in this study experienced mild COVID-19 infection,^30^ was vaccinated against COVID, and did not report persistent symptoms following SARS-CoV-2 infection.

Even at study entry, several findings specific to infected compared to uninfected participants are intriguing and warrant closer monitoring. The presence of non-specific gastrointestinal symptoms appears to be a unique feature of pediatric infected participants.^31,32^ While there have been a few case reports of children developing inflammatory bowel disease following SARS-CoV-2 infection,^33,34^ we did not observe a trend of increased new gastrointestinal new medical diagnosis in our infected cohort.

The report of persistent fatigue and shortness of breath did not correlate with radiological findings or abnormalities on the cardiopulmonary evaluation; however, older adolescent infected participants (>16 years) had a worse performance on the six-minute walk test compared to their aged uninfected controls. The 6MWT is simple, age-validated test that can be performed in any clinical setting and has been studied in multiple chronic cardiovascular and pulmonary disease states, as well as in Post COVID conditions, with both adult and pediatric populations, as a surrogate for aerobic activity tolerance (V02 max), endurance, and overall cardiopulmonary fitness.^35,36^ This finding warrants further evaluation as it could be an early indication of decrease endurance or exercise tolerance in this population.

Overall, there were no statistically significant differences observed between cohorts regarding radiographic imaging, pulmonary function (PFTs and DLCO), or cardiac diagnostic findings, however, there were some non-statistically significant trends observed, including higher frequency of ECG ST segment abnormalities and higher incidence of abnormal echocardiogram findings in infected subjects. We found no difference between the 2 groups regarding cardiovascular symptoms including dizziness and fainting. As we evaluate infected participants over a course of 3 years, we will be able to assess if signs of dysautonomia develops over time^37^ or if there are changes in the cardiac function in children recovering from acute COVID-19.^38^

Baseline psychosocial, quality of life and developmental survey data in our study also highlight the importance of inclusion of an uninfected participants for comparison. While some studies have suggested symptoms of anxiety and depression as a feature of pediatric PACS/Long COVID,^39,40^ this study demonstrates that anxiety, depressive symptoms, and difficulties with peer relationships were reported by parents and participants in a large and similar proportion in both cohorts, thus reflecting the potential consequences of social isolation related to the pandemic. In contrast, other psychosocial domains revealed worsening symptoms unique to infected participants, including the domains of Global Health, Physical Functioning and Mobility, Cognitive Functioning, Pain Interference, Fatigue, and Sleep Disturbances. The design of this study allows a more refined understanding of psychosocial and developmental impacts specific to SARS-CoV-2 infection, in contrast to more general effects on children as a result of living through the turbulent pandemic and post-pandemic period.

Of those in the total cohort eligible to receive COVID-19 vaccination, the majority had received at least one vaccination at the time of the baseline visit. We observed that significantly more uninfected subjects received 3 or more doses, when compared to infected. Additionally, most eligible unvaccinated participants were within the infected cohort, suggesting a potential parental/patient perception of decreased urgency for vaccination in this group due to the history of a prior COVID infection.

Despite the many strengths of this study, there are several notable limitations. Participants in this study were self-referred and may result in a selection and reporting bias toward those more focused on the consequences of SARS-CoV-2 infection. Although prospective and longitudinal, the range of time between infection and baseline visit varied, with some infected subjects enrolled more proximal to their infection than others. The median time between infection and baseline visit was 7.3 months. This can be viewed as a limitation, however, it can also be viewed as a window of opportunity to evaluate and compared uninfected and infected cohorts 7 months post infection.

Another potential limitation is that the period of time for which symptoms were reported by uninfected participants (beginning January 2020) was overall longer than the period for infected subjects, who may have been sick anytime between January 2020 and May 2023. This may have resulted in a potential bias in the uninfected group for more symptoms or new conditions to have occurred (as a function of elapsed time), potentially masking any differences between cohorts (underestimating their prevalence in infected or overestimating prevalence in uninfected controls). However, over the full longitudinal follow-up period of 3 years, these variabilities should be minimized. We were unable to perform specific viral sequencing as infected participants enrolled at minimum of 4 weeks after their infection and the majority were PCR negative. However, based on dates of infection, we are able to infer likely infection strain based on US SARS CoV-2 viral surveillance data.

## CONCLUSIONS

We report on the design and characteristics at study entry of a large, well characterized, and prospective cohort of pediatric SARS-CoV-2 infected and uninfected participants to be evaluated longitudinally over 3 years. At study entry, infected subjects were more likely to report constitutional, respiratory, and GI symptoms compared to uninfected participants, and more likely to score worse in validated survey domains of Fatigue, Pain, Global Health, Physical and Cognitive Function, and Sleep disturbances. Both infected and uninfected subjects described increased symptoms of anxiety and depression, most likely a result of living through the turbulent pandemic and post-pandemic period.

Continued systematic longitudinal study of this cohort will help identify clinical sequelae of COVID-19 in children, characterize the immune response to SARS-CoV-2 infection, including the extent and duration of protective immunity, and identify potential genetic/immunologic factors associated with long-term outcomes. Future studies resulting from the study biorepository will provide an opportunity to explore potential pathophysiologic mechanisms of disease and biomarkers of pediatric COVID infection.

## Supplementary Material

Supplemental Material

## Figures and Tables

**Table 1.** Demographics and baseline characteristics including vaccination status, breakthrough infections and reinfection.

	Overall (*n* = 654)	Uninfected (*n* = 113)	Infected (*n* = 541)
Age (Median) (Min,Max)	8.9 (0.6–21.8)	8.9 (0.6–20.8)	8.9 (0.2–21.8)
Age groups (years) % (No.)			
0–1 year	8.6% (56)	7.1% (8)	8.9% (48)
2–4 years	17.0% (111)	13.3% (15)	17.7% (96)
5–11 years	43.1% (282)	49.6% (56)	41.8% (226)
12–17 years	27.1% (177)	27.4% (31)	27.0% (146)
18–21 years	4.3% (28)	2.7% (3)	4.6% (25)
Female % (No.)	47.2% (309)	46.9% (53)	47.3% (256)
Race % (No.)			
White	55.8% (365)	68.1% (77)	53.2% (288)
Black	25.2% (165)	14.2% (16)	27.5% (149)
More than one	10.1% (66)	8% (9)	10.5% (57)
Asian	2.1% (14)	2.7% (3)	2% (11)
American Indian	1% (3)	0%	1% (3)
Ethnicity % (No.)			
Hispanic	13.8% (90)	8.8% (10)	14.8% (80)
Pre-existing medical conditions % (No.)	59% (386)	55.8% (63)	59.7% (323)
Second hand smoking exposure % (No.)	6.7% (44)	5.3% (6)	7% (38)
Median months from COVID-19 symptoms to Baseline visit (Q1-Q3)^a^			7.3(4.1–12.3)
Median months from COVID-19 testing to Baseline visit (Q1-Q3)^b^			6.7 (3.5–11.6)
Not age eligible for SARS-CoV2 vaccination at Baseline^c^ % (No.)	17.1% (112)	16.8% (19)	17.2% (93)
Eligible for SARS-CoV-2 vaccination^c^ % (No.)	82.9% (542)	83.2% (94)	82.8% (448)
Received 3 or 4 vaccine doses prior to Baseline	41.2% (223/542)	51.1% (48/94)	39.1% (175/448)
Received 2 vaccine doses prior to Baseline	39.7% (215/542)	42.6% (40/94)	39.1% (175/448)
Received 1 vaccine dose prior to Baseline	3.1% (17/542)	1.1% (1/94)	3.6% (16/448)
Received 0 vaccine dose prior to Baseline	15.3% (83/542)	5.3% (5/94)	17.4% (78/448)
Proportion of first infections being a breakthrough infection (%)^d^ % (No.)			53.6% (149/278)
SARS-CoV-2 reinfection % (No.)			11.1% (60/541)
Severity of reinfection (Mild)^e^ % (No.)			91.5% (54/59)
Proportion of participants who required a hospitalization after recovering from initial SARS-CoV-2 infection (%)^f^			4.6% (25/541)

*COVID-19* Coronavirus Disease 2019, *Q1* Quartile 1, *Q3* Quartile 3.

a Among symptomatic infected participants.

b Among asymptomatic infected participants.

c SARS-COV-2 vaccine eligibility was considered depending on age and status of Emergency Use Authorization (EUA) by the FDA at baseline. All participants (infected and uninfected) age 12+ were considered eligible at the start of the study. Participants 5–12 years became eligible on October 29, 2021, and participants 6 months to 4 years became eligible on June 17, 2022. Infants 0–6 months of age were not eligible for vaccination.

d Breakthrough infections at baseline were defined when the COVID-19 vaccine regimen completion date was at least 14 days prior to the earliest of 1.) COVID-19 symptoms onset; 2.) specimen collection date of SARS-CoV-2 positive PCR; or 3.) specimen collection date of COVID-19 positive antigen test whichever happened first. Vaccine regimen completion date was defined as either the date of the first Johnson & Johnson vaccine, or the second vaccine of another type. Only 278 infected participants were considered to have completed a vaccine regimen.

e 54 (92%) infected participants reported having a mild infection, 3 participants reported a moderate infection^22^ and 2 participants reported a severe infection.^22^ Severity of reinfection was unknown for 1 participant.

f 25 infected participants required hospitalization after recovering from the initial episode of SARS-CoV-2 infection. Two infected participants had hospitalizations (stroke (*n* = 1) and hypoxia (*n* = 1)) documented to be related to SARS-CoV-2 infection, 2 other participants had psychiatric hospitalizations with temporal association to SARS-CoV-2 infection (worsening of depression requiring inpatient admission (*n* = 1), new onset suicidal ideation (*n* = 1)). Other hospitalizations were related to infections (*n* = 7), surgical procedures (*n* = 5), psychiatric symptoms (*n* = 1), neurological symptoms (*n* = 2) and respiratory symptoms (*n* = 2). There were single admissions related to a flare of an autoinflammatory disease, anemia, Kawasaki-like disease and concern for MIS-C which was ruled out.

**Table 2.** Comparison of PROMIS domain T-scores between infected and uninfected – parent and child report.

PROMIS measure	Uninfected mean (SD)	Infected mean (SD)	Adjusted *p*-value^a^	Uninfected mean (SD)	Infected mean (SD)	Adjusted *p*-value^a^
	Parent Reported (all children)			Child Reported (Age 8+)		
Global Health ↑	51.50 (8.51) No = 104	48.43 (10.15) No = 487	**0.004**	48.39 (8.66) No = 61	46.91 (8.88) No = 271	0.21
Physical Functioning ↑	52.50 (6.83) No = 86	49.80 (8.29) No = 365	**0.005**	52.19 (7.73) No = 64	50.72 (9.02) No = 295	0.24
Cognitive Functioning ↑	51.02 (8.96) No = 86	47.98 (8.90) No = 351	**0.006**	47.99 (8.30) No = 64	46.59 (7.79) No = 291	0.19
Pain Interference ↓	43.26 (8.58) No = 86	48.08 (11.33) No = 361	**0.001**	40.92 (9.22) No = 64	44.49 (10.49) No = 293	**0.008**
Fatigue ↓	40.69 (9.56) No = 86	46.83 (12.91) No = 363	**<.001**	43.13 (10.95) No = 64	47.35 (12.64) No = 292	**0.01**
Anxiety ↓	52.28 (9.98) No = 104	51.94 (9.70) No = 486	0.56	50.58 (9.14) No = 64	49.60 (10.01) No = 294	0.51
Social Relationships ↑	53.41 (7.37) No = 18	51.92 (7.85) No = 122	0.71	N/A	N/A	N/A
Peer Relationships ↑	50.83 (10.76) No = 86	48.55 (10.83) No = 362	0.10	47.99 (9.54) No = 61	47.91 (10.12) No = 268	0.90
Sleep Disturbance ↓	51.92 (9.92) No = 104	54.38 (10.62) No = 481	**0.02**	50.89 (9.87) No = 64	53.25 (10.53) No = 291	0.11
Depressive Symptoms ↓	49.10 (8.89) No = 104	49.93 (8.55) No = 486	0.47	50.34 (9.17) No = 64	49.56 (10.12) No = 293	0.54

Results are shown in mean and standard deviations for infected and uninfected participants. Up arrow indicates domains in which higher T score is better performance. Down arrow indicates domains in which lower T score indicates better performance.

*PROMIS* Patient-Reported Outcomes Measurement Information System, *SD* Standard deviation, *GEE* Generalized Estimated Equations, *N/A* not available.

a *p*-values are computed using linear models comparing mean results between infected and uninfected, adjusting for age and sex and estimated using generalized estimated equations to account for correlation between family members.

## Data Availability

The datasets generated during and or analyzed during the current study are not publicly available due as the study is currently in progress and the datasets are continuously updated. Individual patient/participant data will not be provided at the time of publication.

## IMPACT:

- This study establishes a large, diverse, prospective, longitudinal, multi-center cohort of children with history of SARS-CoV-2 infection compared to an uninfected cohort to be followed for 3 years.
- Cross-sectional cohort analysis at study entry showed infected participants were more likely to report constitutional, respiratory, and GI symptoms compared to uninfected controls.
- Infected participants were more likely to have significantly worse parent-reported performance in 6 of 10 Patient Reported Outcome Measures domains.
- Continued study of this cohort will help identify clinical sequelae of COVID-19, characterize the immune response to SARS-CoV-2 infection, and identify potential genetic/immunologic factors associated with long-term outcomes.
