# Supplementary material for: Pediatric SARS-CoV-2 long term outcomes study (PECOS): cross sectional analysis at baseline
Source: Pediatr Res. 2024 Dec 18;98(2):541–50. doi: 10.1038/s41390-024-03777-1 (PMC12174577; doi:10.1038/s41390-024-03777-1)
Supplement: Supplementary file 1 — Online Material_ Pediatric Research_R3_CLEAN [file 41390_2024_3777_MOESM1_ESM.pdf]

eMETHODS:

Study objectives
Study Visits
eFigure 1. Protocol Schema
eTable 1. Schedule of baseline activities for Infected and Uninfected Participants
Data Collection
Demographics
Vital signs
Research Samples
Radiological Imaging
Pulmonary Function Tests (PFTs)
6MWT
Diffusion capacity of the lungs for carbon Monoxide (DLCO)
Echocardiogram and electrocardiogram
Cardiac MRI
Questionnaires
eTable 2. World Health Organization (WHO) Case Report Form (CRF) for incidence of symptoms after COVID-19

eRESULTS

eTable 3. Frequency of Medical Conditions prior to 2020
eFigure 2. Enrollment Distribution by Variant Wave of SARS-CoV-2 Infected Participants
Baseline Visit Assessments
eFigure 3. Comparison of Review of Systems Symptom Clusters: Infected vs. Uninfected
eFigure 4. Comparison of Physical Examination Abnormalities: Infected vs. Uninfected
eFigure 5. Comparison of Abnormal Chest CT or Chest Radiography: Infected vs. Uninfected
eTable 4. Comparison of Specific Radiographic Abnormalities: Infected vs. Uninfected
eTable 5. Comparison of Abnormal Spirometry, Lung Volume and Diffusion Lung Capacity: Infected vs. Uninfected
eTable 6. Comparison of Pulmonary Spirometry, Lung Volumes and Diffusion Lung Capacity: Infected vs. Uninfected
eTable 7. Comparison of 6 Minute Walk Test: Infected vs. Uninfected (Participants ≥ 5 years of age)
eTable 8. Comparison of Distance walked on 6 Minute Walk Test: Infected vs. Uninfected
eTable 9. Comparison of Electrocardiogram Findings: Infected vs. Uninfected
eTable 10. Comparison of Echocardiogram Findings: Infected vs. Uninfected
eTable 11. Comparison Coronary Artery Z-score by Echocardiography: Infected vs. Uninfected
eTable 12. PROMIS -Participants Identified to have a Clinically Elevated Scores (≥1 Standard Deviation (SD) worse T score) Across Domains between Infected and Uninfected Participants - Parent and Child Report
eFigure 6. Parent-Reported PROMIS Domains with T-Scores Significantly Different Comparing Infected to Uninfected Participants
eTable 13. Comparison of CRISIS composites (generalized worry vs. worry about COVID) between Infected and Uninfected Participants - Parent and Child Reports
eTable 14. Laboratory Results Outside Pediatric Reference Range for Age and Sex among Infected vs. Uninfected Participants

eTable 15. Comparison of Laboratory Results Outside Pediatric Reference Range for Age and Sex among Infected Participants Evaluated < than 180 Days from First SARS-CoV-2 Infection vs. ≥ 180 Days
eTable 16. Comparison SARS-CoV-2 PCR, Anti-nucleocapsid and Total Neutralizing Antibodies between Infected and Uninfected Participants
eFigure 7. Comparison of SARS-CoV-2 Total Neutralizing Antibody with Vaccination Status between Infected and Uninfected Participants

Supplement Material - Pediatric SARS-CoV-2 Long-term Outcomes Study (PECOS): Cross Sectional Analysis at Baseline

eMETHODS:

Primary objectives: To describe findings at study entry from an ongoing longitudinal study aimed to characterize the long-term clinical manifestations and sequelae following recovery from SARS-CoV-2 infection.

Secondary objectives:

Determine whether there are genetic factors influencing long-term outcomes.

Characterize changes in the SARS-CoV-2 immune response over time.

Determine whether there are immunological factors influencing long-term outcomes.

Study Visits:

1. **Study schedule**: Upon enrollment, infected participants agreed to be evaluated every 6 months for 3 years. Infected participants who enrolled within 12 weeks of their initial infection underwent an extra protocol visit at 3-months. Uninfected participants agreed to be seen annually for 3 years. (eFigure 1)

eFigure 1. Protocol Schema

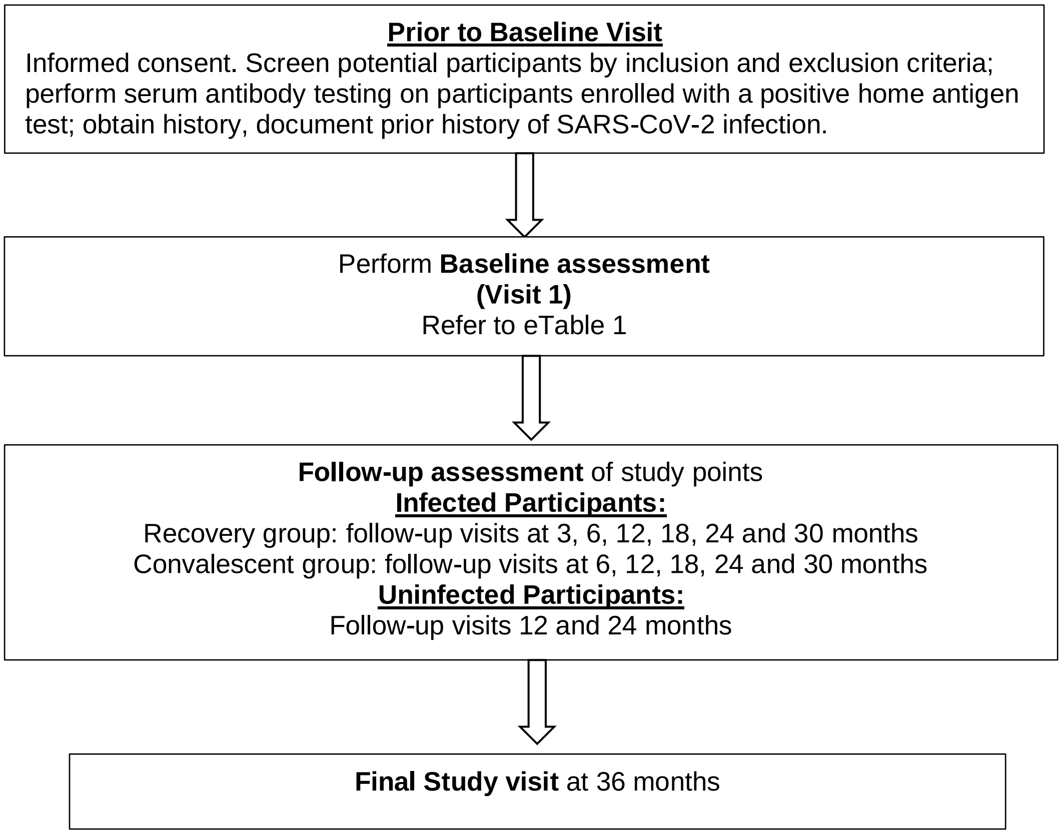

eTable 1. Schedule of baseline activities for Infected and Uninfected Participants

Procedures	Screening Day -13 to 1	Baseline Day 1
Informed consent	X	
Demographics	X	
Medical history	X	X
Concomitant medication review	X	X
Physical exam (including capillaroscopy, neurological exam)		X
Vital signs		X
Height		X
Weight		X
Developmental history form		X
PROMIS Global Functioning + Supplemental Measures		X
Developmental screener		X
CRISIS questionnaire-initial		X
SARS-CoV-2 RNA testing [a]		X
SARS-CoV-2 antibodies [a]		X
Hematology [b]		X
Serum chemistry [c]		X
Urinalysis		X
Human chorionic gonadotropin [d]		X
Echocardiogram		X
Electrocardiogram		X
Cardiac MRI (without contrast)		X
Pulmonary Function Test		X
Low-radiation chest CT or AP chest radiograph (without contrast)		X
Research blood sample for genetics (optional)		X
Research blood samples for immunology/inflammatory assays		X
Research stool		X
Adverse Events review and evaluation		X
Complete eCRFs	X	X
Clinical photography (optional)		X

[a:] Emergency Use Authorization (EUA)–approved SARS-CoV-2 assays

[b:] white blood cells (WBC), red blood cells (RBC), hemoglobin, hematocrit, mean corpuscular volume (MCV), mean corpuscular hemoglobin (MCH), mean corpuscular hemoglobin concentration (MCHC), red blood cell distribution width (RDW), platelets, mean platelet volume (MPV), nucleated RBC, neutrophils, lymphocytes, monocytes, eosinophils, basophils, D-dimer, fibrinogen, erythrocyte sedimentation rate (ESR)

[c:] sodium (Na), potassium (K), chloride (Cl), total carbon dioxide (CO_2, bicarbonate), creatinine, glucose, urea nitrogen, estimated glomerular filtration rate (eGFR), anion gap, alkaline phosphatase, alanine aminotransferase (ALT)/glutamate-pyruvate transaminase (GPT), aspartate aminotransferase (AST)/glutamic oxaloacetic transaminase (GOT), total bilirubin, direct bilirubin, total cholesterol, triglycerides, high-density lipoprotein (HDL) cholesterol and low density lipoprotein (LDL cholesterol (calculated), troponin I, pro-brain natriuretic peptide (proBNP), creatine kinase (CK), lactate dehydrogenase (LDH), ferritin, C3 and C4, C-reactive protein (CRP), thyroid stimulating hormone (TSH), and free T4

[d:] to be done prior to chest CT for participants of childbearing potential.

Abbreviations: AP (anterior-posterior); CRISIS (CoRonavIruS health Impact Survey); CT (computed tomography); eCRF, (electronic case report form); MRI (magnetic resonance imaging); PROMIS (Patient Reported Outcomes Measurement Information System); SARS-CoV-2 (severe acute respiratory syndrome coronavirus 2).

Data Collection:

1. **Demographics**: Age, sex, self-reported race and ethnicity, insurance type and zip code were included.

2. **Vital signs:** Body temperature, blood pressure (measured on the right arm [preferably] and using an age and size appropriate cuff bladder), heart rate, respiratory rate, and oxygen saturation were collected.

3. **Research Samples:** Nasopharyngeal (NP) swab and stool samples were assessed for persistent viral DNA using SARS-CoV-2 PCR. Blood samples were evaluated for complete blood cell count (CBC), routine renal and liver function, inflammatory markers, fibrinogen, d-dimer levels, thyroid function, troponin I, pro-B-type natriuretic peptide (Pro-BNP), lipid panel, C3 and C4; urinalysis was also obtained. Research samples included DNA, PAXgene, and serum tubes. SARS-CoV-2 antibodies included Biorad Platelia SARS-CoV-2 Total Antibody and GenScript SARS-CoV-2 Neutralization Antibody.

   - Biorad Platelia SARS-CoV-2 Total Ab Assay: One-step antigen capture format Enzyme-Linked Immunosorbent Assay (ELISA) for qualitative detection of total anti-SARS-CoV-2 nucleocapsid antibodies (IgM/IgA/IgG) in human serum or plasma specimens. Normal Value = Negative

   ● GenScript cPassTM SARS-CoV-2 Neutralization Antibody Detection Kit: Blocking Enzyme-Linked Immunosorbent Assay (ELISA) intended for qualitative direct detection of total neutralizing antibodies to SARS-CoV-2 in human serum and K2-EDTA plasma. Normal Value = Negative.

4. **Radiological imaging:** chest computed tomography (CT), or chest radiograph (CXR) were attempted in all participants based on parental preference, and following Standard of Operation Procedures (SOPs). Images were reviewed by an adult radiologist at NIH and a group of pediatric radiologists at CNH. All radiologists were blinded to the participants status cohort.

5. **Pulmonary Function Tests (PFTs):** Procedures were attempted in participants aged 5 years and older, unless the participant had a clinical/developmental contraindication. For participants aged 5 to 6 years, PFTs included spirometry, lung volumes, and 6-minute walk test (6MWT). For participants aged 7 years and older, additional diffusing capacity for carbon monoxide (DLCO) was performed. PFTs, 6MWT and DLCO results were reviewed by a blinded pulmonologist.

   PFTs were completed and reported according to the American Thoracic Society (ATS) and European Respiratory Society Technical Statement (ERS). Lung volumes were measured with the Multiple Breath Washout technique (aka Nitrogen Washout) which also calculated Lung Clearance Index (LCI)[1]. If

clinically indicated, bronchodilators were used, and response assessed according to the ATS/ERS guidelines.

The Global Lung Initiative (GLI)[2] calculations were used for predicted normal values using race-neutral equations. Calculations not available at the GLI site, were not included (I.E. FEF 25-75).

6. **6MWT:** was performed according to the ATS/ERS guidelines[3]. Under direct observation and monitoring of the Heart Rate, Respiratory Rate and Oxyhemoglobin saturation. The participant's age, sex, race, height, and weight were recorded as well as the total distance walked.
7. **Diffusion capacity of the lungs for carbon Monoxide (DLCO)**: Participants 7 years of age and older had additional testing for DLCO[4], unless clinically contraindicated. DLCO was calculated using adjusted hemoglobin values obtained within a month from the procedure. GLI calculations were done for predicted normal values.
8. **Echocardiogram and electrocardiogram** were attempted in all participants under specific SOPs at both institutions. All echocardiographic images were transferred to CNH for a centralized review which was completed by a blinded pediatric cardiologist. Abnormal echocardiogram was defined as: the presence of at least one of the following: (1) Myocardial systolic dysfunction if ejection fraction was < 55%[5] or shortening fraction < 27[6], (2) coronary artery abnormalities defined as (a) dilation (Z-score 2 to 2.4)[7], (b) small aneurysm (Z-score 2.5 to 4.9), (3) more than trivial pericardial effusion, (4) atrioventricular valve regurgitation defined as > trivial mitral valve regurgitation and/or > mild tricuspid valve regurgitation, and/or (5) > trivial aortic valve insufficiency [8].
9. **Cardiac MRI**: first cardiac MRI was offered at least 6 weeks after onset of symptoms. This procedure was only offered to all participants 8 years of age or older. Participants requiring conscious sedation were excluded from this procedure.
10. **Questionaries:**
    a. **Developmental history form** was completed by the investigator or parent/guardian via the Research Electronic Data Capture system (REDCap). If there was no parent/guardian available for a participant ≥ 18 years, the form was not completed.
    b. Patient Reported Outcomes Measurement Information System (PROMIS): The PROMIS tools were developed by NIH and have excellent psychometric properties[9]. For all PROMIS measurements

data was collected via REDCap. Data was provided by guardian-report for participants aged 1 to 17 years, child self-report for participants 8 to 17 years, and adult self-report for participants ≥ 18 years. Global functioning was assessed using the PROMIS Pediatric/Parent Proxy Profile (25 items) or comparable adult self-report (for participants ≥ 18 years), assessing functioning in the following areas: physical function mobility, anxiety, depressive symptoms, fatigue, peer relationships, pain interference, and pain intensity. For supplemental measures refer to sleep disturbance, global health, and cognitive impact. PROMIS questionnaires can be found at: https://www.healthmeasures.net/

c. **Developmental Profile-4 (DP4):** The DP4 survey was included to identify developmental and strengths and weaknesses in five key areas. This survey was completed by guardians of participants aged 0 to 17 years in REDCap.

d. **CoRonavIruS health Impact Survey (CRISIS):** The CRISIS Survey was designed to enable researchers to examine the extend and impact of life changes induced by the COVID-19 epidemic on the mental health, and behavior of individuals and families across diverse international settings[10]. For CRISIS measurements, guardians of participants ≥ 5 years and participants ≥ 11 years were asked to complete this survey via REDCap. The initial guardian-report survey completed at baseline included additional items from the CRISIS AFAR (adapted for autism and related neurodevelopmental conditions) and CRISIS Adult Self-Report. The surveys can be found at: http://www.crisissurvey.org/download/.

eTable 2. World Health Organization (WHO) Case Report Form (CRF) for incidence of symptoms after COVID-19

The data for this CRF was collected as follows: Infected participants were asked if any of the symptoms included in the CRF have been present after recovering from COVID. Uninfected controls were asked if any of the symptoms were present over the last 6-12 months or at the time of the baseline visit. Responses were categorized as never present, present in the past but currently resolved, or present at the time of the visit (intermittent vs. persistent). All answers were collected and not adjusted for pre-existing medical conditions prior to January 2020.

**Symptoms after COVID-19**
Anxiety
Behavior change
Can’t move and/or feel one side of body or face
Chest pain
Constipation
Depressed mood
Diarrhea
Dysmenorrhea
Dizziness/light headedness
Fainting/blackouts
Fever (persistent)
Forgetfulness
Jerking of limbs
Joint pain/swelling
Loss of appetite
Loss of interest/pleasure
Lumpy lesions: (purple/pink/bluish) on toes/COVID toes
Nausea/vomiting
Numbness or tingling
Pain on breathing
Palpitations
Persistent dry cough
Persistent fatigue
Problems hearing
Persistent headache
Persistent muscle pain
Post-exertional malaise
Problems passing urine
Problems seeing
Problem swallowing
Problems with balance
Problems with gait/falls
Reduced smell
Reduced taste
Ringing in ears
Seizures
Shortness of breath: If yes: Present At rest? With activity?
Skin rash: If yes, please tick all areas of the body that apply: Face / Trunk (stomach or back) / Arms / Legs / Buttocks / Toes / Fingers;
Slowness of movement
Sleeping less
Sleeping more
Stiffness of muscles
Stomach pain
Swollen ankles
Tremors
Trouble in concentrating
Weakness in limbs
Weight loss
Erectile dysfunction
Hallucinations

eRESULTS:

eTable 3. Frequency of Medical Conditions prior to 2020

Medical Conditions prior to 2020	Overall (No=654)	Uninfected (No=113)	Infected (No=541)
Asthma	15.3% (100)	13.3% (15)	15.7% (85)
Neurodevelopmental	12.5% (82)	9.7% (11)	13.1% (71)
Psychiatric	12.2% (80)	11.5% (13)	12.4% (67)
Allergy	8.1% (53)	9.7% (11)	7.8% (42)
Prematurity	6.4% (42)	3.5% (4)	7% (38)
Neurological	5.2% (34)	5.3% (6)	5.2% (28)
Gastrointestinal	4.4% (29)	4.4% (5)	4.4% (24)
Cardiovascular	3.4% (22)	1.8% (2)	3.7% (20)
History of adenoidectomy / tonsillectomy	3.1% (20)	3.5% (4)	3% (16)
Hematology	1.8% (12)	0.9% (1)	2% (11)
Endocrinology	1.7% (11)	2.7% (3)	1.5% (8)
Obesity	1.7% (11)	0.9% (1)	1.8% (10)
Other[a]	16.4% (107)	14.2% (16)	16.8% (91)

[a] Mostly related to dermatological pre-existing conditions and or elective surgeries.
Less than 1% of participants (not included in this table) reported history of autoimmunity (n=5), oncologic disorders (n=3), diabetes (n=4), liver disease (n=2), immunodeficiency (n=1), hypertension (n=1), Kawasaki Disease (n=1). The presence of underlying medical conditions was very similar among both cohorts (Table 1).

eFigure 2. Enrollment Distribution by Variant Wave of SARS-CoV-2 Infected Participants

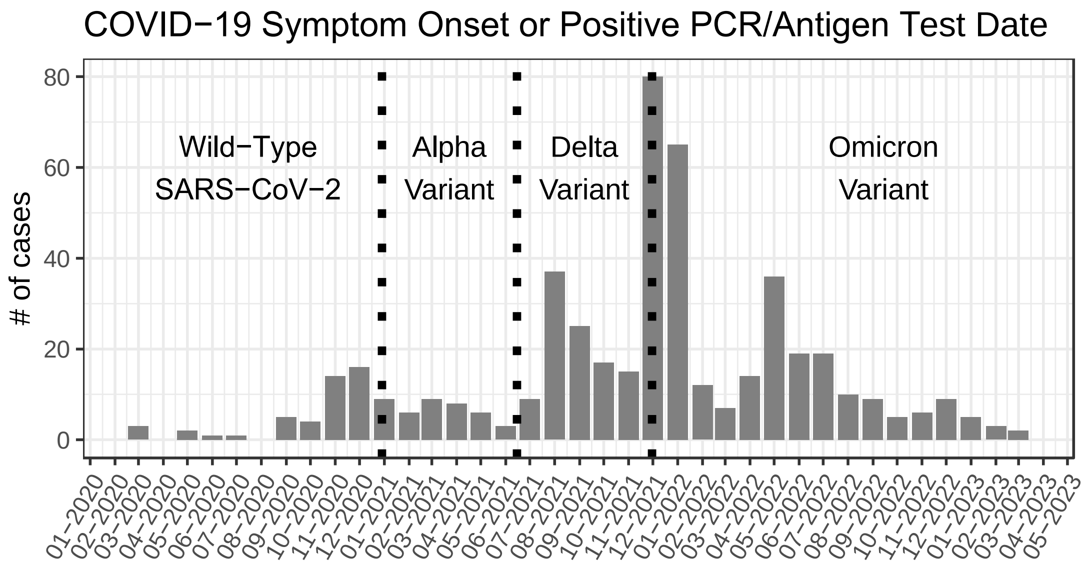

Distribution of enrollment of infected participants by variant wave using as a reference the date of symptom onset for symptomatic infected participants and positive SARS-CoV-2 PCR or laboratory-confirmed antigen for asymptomatic infected participants. This diagram does not include infected participants identified by positive anti-SARS-CoV-2 nucleocapsid antibody. Number of COVID-19 cases (y-axis) are represented over time (x-axis). Dotted lines represent time of transition among predominant variants. Most infected participants were recruited over the Omicron Variant period.
Abbreviations: PCR (polymerase chain reaction).

Baseline Visit Assessments

eFigure 3. Comparison of Review of Systems Symptom Clusters: Infected vs. Uninfected

System	Uninfected	Infected	Odds Ratio	95% CI	p–value
Any System	62.8% (71/113)	71.2% (385/541)	1.59	(1.02, 2.50)	0.04
Constitutional	20.4% (23/113)	32.0% (173/541)	2.24	(1.36, 3.67)	0.001
HEENT	2.7% (3/113)	9.1% (49/541)	3.74	(1.12, 12.48)	0.03
Cardiovascular	14.2% (16/113)	19.2% (104/541)	1.54	(0.75, 3.16)	0.24
Respiratory	8.8% (10/113)	18.3% (99/541)	2.41	(1.19, 4.88)	0.01
GI	22.1% (25/113)	40.5% (219/541)	2.58	(1.64, 4.07)	< 0.001
GU	4.4% (5/113)	7.2% (39/541)	1.64	(0.56, 4.81)	0.37
Musculoskeletal	9.7% (11/113)	11.3% (61/541)	1.17	(0.56, 2.43)	0.67
Neurologic	28.3% (32/113)	36.6% (198/541)	1.58	(0.98, 2.54)	0.06
Psychiatric	39.8% (45/113)	38.9% (210/540)	0.93	(0.60, 1.45)	0.77
Skin or Tegumentary	13.3% (15/113)	10.4% (56/541)	0.76	(0.42, 1.36)	0.35

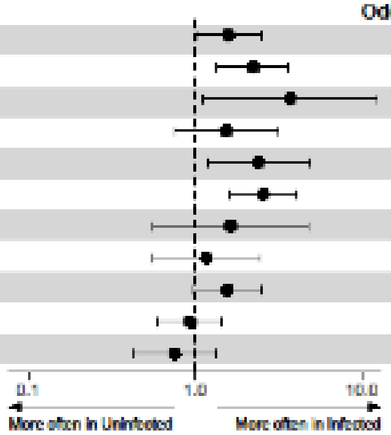

Percentage of participants reporting symptoms since recovery of SARS-CoV-2 infection (Infected) or since 2020 (uninfected), with odds ratio reporting the odds of infected participants reporting symptoms comparing to uninfected. p-values comparing odds of the findings between infected and uninfected participants are adjusted for age and sex. Generalized estimating equations (GEE) are used to account for correlation between family members participating in the study.
Abbreviations: CI (confidence interval), GI (gastrointestinal), GU (genitourinary), HEENT (head, ear, nose and throat).

eFigure 4. Comparison of Physical Examination Abnormalities: Infected vs. Uninfected

System	Uninfected	Infected	Odds Ratio	95% CI	p–value
General	0.9% (1/113)	1.8% (10/541)	2.09	(0.27, 16.35)	0.48
Skin	7.1% (8/113)	6.5% (35/541)	1.31	(0.47, 3.67)	0.61
Head	0.0% (0/113)	0.6% (3/540)	Inf	(0.09, Inf)	> 0.99
Eyes	1.8% (2/113)	1.1% (6/539)	0.63	(0.13, 3.11)	0.57
Ears	1.8% (2/111)	1.9% (10/537)	1.02	(0.22, 4.64)	0.98
Nose	1.8% (2/113)	0.9% (5/537)	0.54	(0.11, 2.75)	0.46
Oral cavity	0.9% (1/113)	2.0% (11/538)	2.99	(0.18, 49.26)	0.44
Neck	0.0% (0/113)	0.6% (3/540)	Inf	(0.09, Inf)	> 0.99
Lymph Nodes	6.2% (7/113)	9.8% (53/540)	1.79	(0.66, 4.80)	0.25
Pulmonary	0.0% (0/113)	1.1% (6/540)	Inf	(0.24, Inf)	0.64
Cardiovascular	1.8% (2/113)	2.6% (14/540)	1.56	(0.38, 6.44)	0.54
Abdominal	0.9% (1/113)	0.4% (2/541)	0.41	(0.04, 4.43)	0.47
Musculoskeletal	1.8% (2/113)	2.6% (14/539)	2.02	(0.48, 8.46)	0.33
Neurologic	0.0% (0/113)	3.0% (16/538)	Inf	(0.82, Inf)	0.09

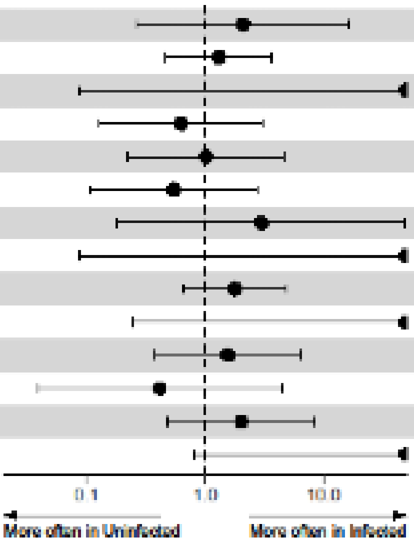

Percentage of participants reported to have abnormal findings on physical exam at the time of the baseline visit, with odds ratio reporting the odds of infected participants having an abnormal finding on physical exam comparing to uninfected. p-values comparing odds of the findings between infected and uninfected are adjusted for age and sex. Generalized estimating equations (GEE) are used to account for correlation between family members participating in the study. Abbreviations: CI (confidence interval).

eFigure 5. Comparison of Abnormal Chest CT or Chest Radiography: Infected vs. Uninfected

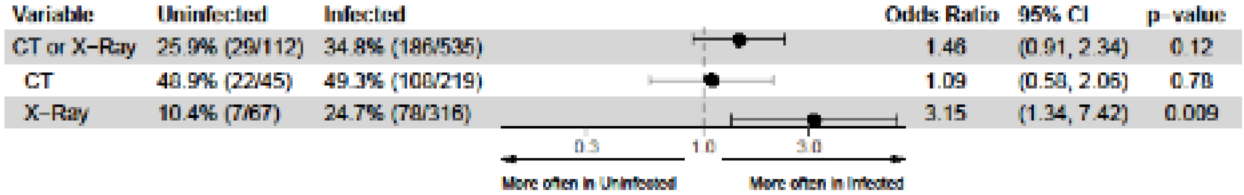

Percentage of participants having abnormal imaging results, with odds ratio of abnormal results comparing infected participants to uninfected. p-values comparing odds of the findings between infected and uninfected are adjusted for age and sex. Generalized estimating equations (GEE) are used to account for correlation between family members participating in the study
Abbreviations: CI (confidence interval), CT (Computed tomography).

eTable 4. Comparison of Specific Radiographic Abnormalities: Infected vs. Uninfected

Procedure	Findings	Uninfected (No=112)	Infected (No=535)	Total	Odds Ratio	95% CI	p-value
CT	Ground glass opacities	4.4% (2/45)	5.5% (12/220)	5.3% (14/265)	1.43	(0.35, 5.83)	0.62
CT	Consolidation	0.0% (0/45)	0.5% (1/220)	0.4% (1/265)	Inf	(0.01, Inf)	>.99
CT	GGO with consolidation	0.0% (0/45)	0.5% (1/220)	0.4% (1/265)	Inf	(0.01, Inf)	>.99
CT	Reticular / Linear opacities	15.6% (7/45)	14.1% (31/220)	14.3% (38/265)	0.91	(0.33, 2.50)	0.85
CT	Nodules	28.9% (13/45)	30.0% (66/220)	29.8% (79/265)	1.02	(0.48, 2.13)	0.96
CT	Bronchial wall thickening	4.4% (2/45)	2.7% (6/220)	3.0% (8/265)	0.60	(0.11, 3.11)	0.54
CXR	Perihilar peribronchial thickening [a]	10.4% (7/67)	19.9% (63/317)	18.2% (70/384)	2.49	(1.03, 6.03)	0.04
CXR	Interstitial pattern	0.0% (0/67)	1.6% (5/317)	1.3% (5/384)	Inf	(0.19, Inf)	0.76
CXR	Opacification/Consolidation	0.0% (0/67)	1.6% (5/317)	1.3% (5/384)	Inf	(0.19, Inf)	0.76
CXR	Ground Glass opacities	0.0% (0/67)	0.0% (0/317)	0.0% (0/384)	1.00	(0.00, Inf)	>.99
CXR	Pleural effusions	0.0% (0/67)	0.6% (2/317)	0.5% (2/384)	Inf	(0.04, Inf)	>.99
CXR	Other X-Ray abnormal finding	3.0% (2/67)	3.5% (11/317)	3.4% (13/384)	1.11	(0.24, 5.10)	0.90

Percentage of participants having abnormal imaging findings, with odds ratio of abnormal results comparing infected participants to uninfected. p-values comparing odds of the findings between infected and uninfected are adjusted for age and sex. Generalized estimating equations (GEE) are used to account for correlation between family members participating in the study.
Abbreviations: CI (confidence interval), CT (Computed tomography), CXR (Chest X-ray), GGO (Ground glass opacities).
[a] Perihiliar peribronchial thickening was present in 70 participants of those, 20 participants (19 infected and 1 uninfected) were documented to have pulmonary pathology by medical history. Of the infected participants, 7/19 had recent respiratory infections, 7 had history of asthma, 3 of bronchopulmonary dysplasia, 1 cystic fibrosis and 1 pulmonary vein stenosis. The only uninfected participant with presence of peribronchial thickening had history of a recent croup infection.

eTable 5. Comparison of Abnormal Spirometry, lung volume and Diffusion Lung Capacity: Infected vs. Uninfected

Variable	Uninfected (%) abnormal (No=90)	Infected (%) abnormal (No=397)	Odds Ratio	95% CI	p-value
FVC below LLN	2.3% (2/87)	5.7% (21/370)	2.87	(0.65, 12.67)	0.16
FEV1 below LLN	6.9% (6/87)	6.5% (24/370)	0.94	(0.37, 2.40)	0.90
FEV1 / FVC below LLN	13.8% (12/87)	10.0% (37/369)	0.69	(0.34, 1.43)	0.32
TLC below LLN	6.1% (4/66)	2.4% (7/287)	0.41	(0.11, 1.51)	0.18
FRC below LLN	13.6% (9/66)	10.1% (29/288)	0.70	(0.31, 1.58)	0.39
RV above ULN	15.4% (10/65)	9.8% (28/287)	0.62	(0.29, 1.33)	0.21
RV / TLC above ULN	20.0% (13/65)	15.0% (43/287)	0.75	(0.40, 1.41)	0.37
DLCO below LLN	9.0% (6/67)	6.7% (19/283)	0.82	(0.30, 2.22)	0.70

Results are shown in percentages of infected and uninfected participants. p-values comparing odds of the findings between infected and uninfected are adjusted for age and sex. Generalized estimating equations (GEE) are used to account for correlation between family members participating in the study.
Abbreviations: CI (confidence interval), DLCO (Diffusion Lung Capacity), FEV1 (Forced Expiratory Volume in 1 second), FVC (Forced Vital Capacity), FRC (Functional Residual Capacity), LLN (Lower limit of normal), RV (Residual Volume), TLC (Total Lung Capacity), ULN (Upper limit of normal).

eTable 6. Comparison of Pulmonary Spirometry, Lung Volumes and Diffusion Lung Capacity: Infected vs. Uninfected

Variable	Uninfected Mean (SD); (No=90)	Infected Mean (SD); (No=397)	GEE Coef	95% CI	p-value
FVC (L)	2.67 (1.25)	2.80 (1.24)	-0.07	(-0.21, 0.06)	0.27
FVC % of reference value	104.16 (15.10)	101.65 (19.34)	-2.52	(-6.17, 1.13)	0.18
FVC Z-score	0.31 (1.14)	0.11 (1.40)	-0.19	(-0.47, 0.08)	0.17
FEV1 (L)	2.27 (1.03)	2.40 (1.02)	-0.04	(-0.15, 0.08)	0.56
FEV1 % of reference value	100.88 (16.37)	99.60 (16.63)	-1.01	(-4.89, 2.88)	0.61
FEV1 Z-score	0.09 (1.27)	-0.01 (1.30)	-0.08	(-0.38, 0.22)	0.59
FEV1 / FVC	85.61 (7.42)	86.41 (8.95)	1.17	(-0.52, 2.85)	0.17
FEV1 / FVC % of reference value	96.44 (7.88)	98.02 (8.65)	1.84	(0.09, 3.58)	0.04
FEV1 / FVC Z-score	-0.41 (1.07)	-0.16 (1.20)	0.28	(0.05, 0.52)	0.02
TLC (L)	3.80 (1.63)	4.06 (2.06)	0.04	(-0.27, 0.34)	0.81
TLC % of reference value	110.83 (31.70)	108.39 (34.31)	-2.04	(-10.37, 6.29)	0.63
TLC Z-score	0.72 (2.22)	0.50 (2.06)	-0.19	(-0.76, 0.38)	0.52
FRC (L)	1.92 (1.06)	2.07 (1.59)	0.04	(-0.22, 0.31)	0.75
FRC % of reference value	113.62 (60.46)	109.34 (63.07)	-3.90	(-20.05, 12.24)	0.64
FRC Z-score	0.31 (2.11)	0.13 (1.94)	-0.17	(-0.71, 0.38)	0.55
RV (L)	1.08 (0.83)	1.20 (1.42)	0.07	(-0.18, 0.32)	0.58
RV % of reference value	139.76 (115.30)	136.55 (134.17)	-3.94	(-34.67, 26.80)	0.80
RV Z-score	0.49 (1.67)	0.44 (1.53)	-0.08	(-0.51, 0.34)	0.70

Variable	Uninfected Mean (SD); (No=90)	Infected Mean (SD); (No=397)	GEE Coef	95% CI	p-value
RV / TLC	27.43 (13.53)	27.66 (12.19)	0.34	(-2.92, 3.59)	0.84
RV / TLC % of reference value	130.58 (61.99)	128.30 (55.12)	-1.88	(-16.51, 12.74)	0.80
RV / TLC Z-score	0.64 (1.41)	0.63 (1.25)	-0.01	(-0.35, 0.33)	0.96
LCI (%)	8.76 (2.21)	8.32 (1.98)	-0.11	(-0.70, 0.47)	0.71
DLCO (mL/min/mmHg)	19.75 (6.49)	21.56 (10.03)	0.22	(-2.58, 3.01)	0.88
DLCO % of reference value	105.14 (19.73)	104.22 (21.65)	-0.75	(-6.19, 4.68)	0.78
DLCO Z-score	0.24 (1.21)	0.13 (1.64)	-0.10	(-0.44, 0.25)	0.58

Results are shown in means and Standard Deviation (SD) for infected and uninfected participants. p-values are computed using linear models comparing mean results between infected and uninfected, adjusting for age and sex. Generalized estimating equations (GEE) are used to account for correlation between family members participating in the study.
Abbreviations: CI (confidence interval), Coef (coefficient), DLCO (Diffusion Lung Capacity,) FEV1 (Forced Expiratory Volume in 1 second), FVC (Forced Vital Capacity), FRC (Functional Residual Capacity), LCI (Lung Clearance Index), L (Liter)RV (Residual Volume), Min (Minute), mL (Milliliter), mmHg (Millimeters of mercury), TLC (Total Lung Capacity).

eTable 7. Comparison of 6 Minute Walk Test: Infected vs. Uninfected (Participants ≥ 5 years of age)

Variable	Uninfected Mean (SD); (No=90)	Infected Mean (SD); (No=397)	GEE Coef	95% CI	p-value
Total Distance Covered (Meters)	460.64 (88.43)	469.30 (92.40)	3.63	(-15.53, 22.80)	0.710
Total Distance Covered (Meters)/Height (Meters)	325.55 (61.37)	323.15 (69.24)	1.94	(-12.21, 16.10)	0.788
Borg dyspnea score post-test	1.75 (1.53)	1.55 (1.66)	-0.22	(-0.61, 0.17)	0.264
Oxygen Saturation post-test (%)	96.92 (4.27)	97.21 (3.19)	0.35	(-0.61, 1.30)	0.475

Results are shown in means and Standard Deviation (SD) for infected and uninfected participants. p-values are computed using linear models comparing mean results between infected and uninfected, adjusting for age and sex. Generalized estimating equations (GEE) are used to account for correlation between family members participating in the study.
Abbreviations: CI (confidence interval), Coef (coefficient).

eTable 8. Comparison of Distance walked on 6 Minute Walk Test: Infected vs. Uninfected

Age (years)	Uninfected Mean (SD) (No=85)	Infected Mean (SD) (No=360)	GEE Coef	95% CI	p-value
5 (No=39)	355.36 (85.88)	405.77 (95.12)	52.97	(-5.83, 111.77)	0.08
6-8 (No=104)	467.73 (64.58)	454.78 (74.15)	-17.85	(-45.27, 9.57)	0.20
9-11 (No=113)	445.79 (78.87)	474.88 (95.67)	22.50	(-15.36, 60.36)	0.24
12-15 (No=118)	479.48 (74.01)	497.18 (95.21)	11.61	(-22.94, 46.15)	0.51
≥16 (No=71)	534.56 (92.07)	466.20 (87.33)	-67.31	(-122.58, -12.03)	0.02

Results are shown in means and Standard Deviation (SD) for infected and uninfected participants. p-values are computed using linear models comparing mean results between infected and uninfected, adjusting for age and sex. Generalized estimating equations (GEE) are used to account for correlation between family members participating in the study.
Abbreviations: CI (confidence interval), Coef (coefficient).

eTable 9. Comparison of Electrocardiogram Findings: Infected and Uninfected

Variable	Uninfected (No=113)	Infected (No=533)	p-value
AV Block Abnormality[a]	0 (0.00%)	1 (0.19%)	N/A
Other Cond Abnormality[b]	1 (0.88%)	1 (0.19%)	0.26
Tachycardia Abnormality[c]	0 (0.00%)	0 (0.00%)	N/A
Abnormal Chamber Size[d]	15 (13.27%)	81 (15.20%)	0.48
Abnormal Axis[e]	10 (8.85%)	52 (9.76%)	0.89
Abnormal T Waves[f]	0 (0.00%)	5 (0.94%)	N/A
Abnormal ST segment[g]	0 (0.00%)	1 (0.19%)	N/A
Prolonged QT interval[h]	3 (2.65%)	10 (1.88%)	0.94
Premature ventricular complexes	1 (0.88%)	0 (0.00%)	N/A
Low voltage QRS	0 (0.00%)	0 (0.00%)	N/A
Abnormal ECG Findings	29 (25.66%)	144 (27.02%)	0.68

Results are shown in absolute numbers and percentages for infected and uninfected participants. p-values are computed using linear models comparing mean results between infected and uninfected, adjusting for age and sex. Generalized estimating equations (GEE) are used to account for correlation between family members participating in the study.
[a] 1st degree AV block, 2nd degree AV block, 3rd degree AV block
[b] Right bundle branch block, Left bundle branch block
[c] Ectopic Atrial tachycardia, Supraventricular tachycardia, Ventricular tachycardia
[d] Right atrial enlargement, left atrial enlargement, Left ventricular hypertrophy, right ventricular hypertrophy, Biventricular hypertrophy
[e] Right axis deviation, Left axis deviation, Northwest axis
[f] T wave abnormality, T wave inversion, Flat T waves
[g] ST depression
[h] QTcB > 440 ms (males) and >460 ms (females)
Abbreviations: AV (Atrioventricular), ECG (Electrocardiogram), ms (milliseconds), N/A (not available).

eTable 10. Comparison of Echocardiogram Findings: Infected and Uninfected

Variable	Uninfected Mean (SD) (No=113)	Infected Mean (SD) (No=536)	p-value
Ejection Fraction	63.80 (3.46) No=110	64.16 (3.51) No=527	0.34
Shortening Fraction	36.01 (4.37) No=113	35.88 (4.02) No=533	0.75
BSA (m2)	1.14 (0.47) No=111	1.14 (0.48) No=532	0.49
	No (%) No=113	No (%) No=536	
Myocardial Systolic Dysfunction (No (%))	1 (0.88%) No=113	6 (1.12%) No=536	0.82
Mild	1 (100%) No=1	6 (100%) No=6	
Pericardial Effusion	No=113	No=536	0.59
None	100 (88.50%)	488 (91.04%)	
Trivial	13 (11.50%)	45 (8.40%)	
Small	0 (0.00%)	3 (0.56%)	
Mitral Regurgitation	No=113	No=534	0.51
None	76 (67.26%)	346 (64.79%)	
Trivial	37 (32.74%)	184 (34.46%)	

Variable	Uninfected Mean (SD) (No=113)	Infected Mean (SD) (No=536)	p-value
Mild	0 (0.00%)	4 (0.75%)	
Tricuspid Regurgitation	No=113	No=534	0.07
None	5 (4.42%)	7 (1.31%)	
Trivial	108 (95.58%)	520 (97.38%)	
Mild	0 (0.00%)	7 (1.31%)	
Aortic Regurgitation	No=110	No=530	0.53
None	107 (97.27%)	520 (98.11%)	
Trivial	3 (2.73%)	10 (1.89%)	
Mild	0 (0.00%)	0 (0.00%)	
Abnormal Echocardiogram[a]	1 (0.88%) No=113	17 (3.17%) No=536	0.21

Results are shown in absolute numbers and percentages for infected and uninfected participants. p-values are computed using linear models comparing mean results between infected and uninfected, adjusting for age and sex. Generalized estimating equations (GEE) are used to account for correlation between family members participating in the study.
[a] **Abnormal echocardiogram[8] defined as**: the presence of at least one of the following:

(1) Myocardial systolic dysfunction if ejection fraction was < 55%[5] or shortening fraction < 27[6]
(2) Coronary artery abnormalities defined as (a) dilation (Z-score 2 to 2.4), (b) small aneurysm (Z-score 2.5 to 4.9)
(3) More than trivial pericardial effusion
(4) Atrioventricular valve regurgitation defined as > trivial mitral valve regurgitation and/or > mild tricuspid valve regurgitation.
(5) > trivial aortic valve insufficiency.

Abbreviations: BSA (Body surface area).

eTable 11. Comparison Coronary Artery Z-score by Echocardiography: Infected and Uninfected

Variable	Uninfected Mean (SD) (No=113)	Infected Mean (SD) (No=536)	p-value
LMCA Z score	-0.28 (0.98) No=113	-0.31 (0.84) No=520	0.63
LAD Z score	-0.67 (0.89) No=111	-0.69 (0.82) No=518	0.73
RCA Z score	-0.23 (0.96) No=111	-0.23 (0.85) No=521	0.89
Max Z-score	0.33 (0.80) No=112	0.24 (0.75) No=522	0.23
Z score	No=112	No=522	N/A
Aneurysm	0 (0.00%)	1 (0.19%)	N/A
Dilation	0 (0.00%)	3 (0.57%)	N/A

Results are shown in mean and standard deviation (SD) or percentages for infected and uninfected participants. p-values are computed using linear models comparing mean results between infected and uninfected, adjusting for age and sex and estimated using generalized. Generalized estimating equations (GEE) are used to account for correlation between family members participating in the study.
[a] Coronary artery abnormalities defined as small aneurysm (Z-score 2.5 to 4.9)[7]
[b] Coronary artery abnormalities defined as dilation (Z-score 2 to 2.4)[7]
Abbreviations: LMCA (Left main coronary artery), LAD (Left anterior descending), RCA (Right coronary artery)

eTable 12. PROMIS -Participants Identified to have a Clinically Elevated Scores (≥1 Standard Deviation (SD) worse T score) Across Domains between Infected and Uninfected Participants - Parent and Child Report

PROMIS Measure	Uninfected % at risk	Infected % at risk	Adjusted p-value	Uninfected % at risk	Infected % at risk	Adjusted p-value
	Parent Report			Child Report		
Global Health	10 (9.62%)	89(18.28%)	0.02	12 (19.67%)	53 (19.56)	0.84
Physical Functioning	2 (2.33%)	42 (11.51%)	0.02	4 (6.25%)	35 (11.86%)	0.19
Cognitive Functioning	11 (12.79%)	62 (17.66%)	0.30	10 (15.62%)	54 (18.56%)	0.69
Pain Interference	5 (5.81%)	72 (19.94%)	0.006	3 (4.69%)	23 (7.85%)	0.40
Fatigue	4 (4.65%)	70 (19.28%)	0.003	4 (6.25%)	52 (17.81%)	0.04
Anxiety	20 (19.23%)	102 (20.99%)	0.64	11 (17.19%)	44(14.97%)	0.56
Social Relationships	0 (0.00%)	10 (8.2%)	N/A	N/A	N/A	N/A
Peer Relationships	18 (20.93%)	79 (21.82%)	0.83	11 (18.03%)	60 (22.39%)	0.50
Sleep Disturbance	22 (21.15%)	161 (33.47%)	0.02	14 (21.88%)	80 (27.49%)	0.38
Depressive Symptoms	12 (11.54%)	54 (11.11%)	0.97	9 (14.06%)	48 (16.38%)	0.59

Results are shown in absolute numbers and percentages (%) for infected and uninfected participants. p-values are computed using linear models comparing mean results between infected and uninfected, adjusting for age and sex. Generalized estimating equations (GEE) are used to account for correlation between family members participating in the study.

eFigure 6. Parent-Reported PROMIS Domains with T-Scores Significantly Different Comparing Infected to Uninfected Participants

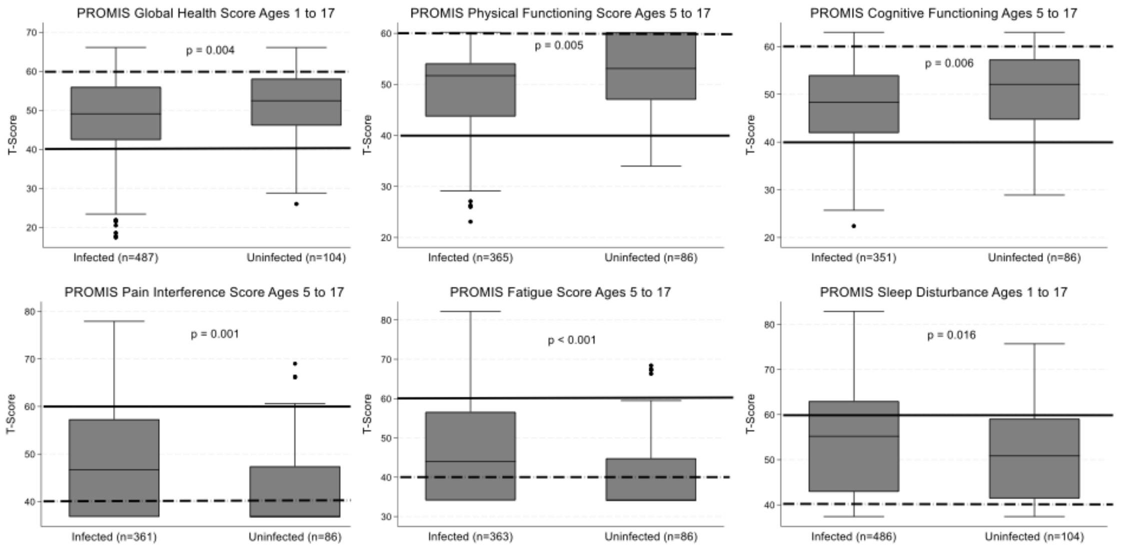

PROMIS Domains in which T scores were statistically different (p<0.05) comparing COVID-19 infected to uninfected participants (Global Health, Physical Functioning, Cognitive Functioning, Pain Interference, Fatigue, and Sleep Disturbance) are depicted graphically. T score of 50 indicates validated and normed median performance in age-matched general population. Dotted line indicates 1SD better performance, and continuous line indicates 1SD worse performance than general population norm. Shaded box indicates cohort IQR; horizontal bar within box indicates median score for cohort

eTable 13. Comparison of CRISIS composites (generalized worry vs. worry about COVID) between Infected and Uninfected Participants - Parent and Child Reports

CRISIS Composites Measure	Uninfected Mean (SD)	Infected Mean (SD)	Adjusted p-value	Uninfected Mean (SD)	Infected Mean (SD)	Adjusted p-value
	All Parent Reports			All Child Reports		
Child COVID Worried	1.74 (0.63) No=87	1.65 (0.69) No=370	0.46	2.11 (0.85) No=33	1.78 (0.70) No=197	0.02
Child Worried	2.01 (0.74) No=87	2.18 (0.74) No=370	0.07	2.56 (0.79) No=33	2.45 (0.75) No=196	0.59
Parent Worried	2.39 (0.56) No=87	2.45 (0.61) No=370	0.99	N/A	N/A	N/A
	Matching Parent Reports			Matching Child Reports		
Child COVID Worried	1.98 (0.67) No=31	1.71 (0.76) No=178	0.26	2.12 (0.87) No=31	1.77 (0.70) No=178	0.02
Child Worried	2.29 (0.89) No=31	2.36 (0.77) No=177	0.38	2.60 (0.78) No=31	2.40 (0.73) No=177	0.26

Results are shown in mean and standard deviations (SD) for infected and uninfected participants. Higher scores indicate worse outcome. p-values are computed using linear models comparing mean results between infected and uninfected, adjusting for age and sex. Generalized estimating equations (GEE) are used to account for correlation between family members participating in the study. Abbreviations: N/A (Not applicable).

eTable 14. Laboratory Results Outside Pediatric Reference Range for Age and Sex among Infected vs. Uninfected Participants.

Variable (units)	Uninfected (% of results above upper limit of normal) n=113	Infected (% of results above upper limit of normal) n-541	Odds Ratio	95% CI	p-value
White Blood Cell Count ($x10^3$/mcL)	5.6% (6/107)	1.8% (9/502)	0.32	(0.12, 0.87)	0.03
Neutrophils (K/mcL)	4.7% (5/107)	1.4% (7/502)	0.28	(0.09,0.90)	0.03
Eosinophils Absolute (K/mcL)	11.2% (12/107)	5.2% (26/502)	0.43	(0.21, 0.86)	0.02

Percentage of participants having abnormal laboratory values. p-values comparing odds of the findings between infected and uninfected participants are adjusted for age and sex. Generalized estimating equations (GEE) are used to account for correlation between family members participating in the study.
Abbreviations: CI (confidence interval), mcL (microliter), K/mcL (thousand cells per cubic millimeter.

eTable 15. Comparison of Laboratory Results Outside Pediatric Reference Range for Age and Sex among Infected Participants Evaluated < than 180 Days from First SARS-CoV-2 Infection vs. ≥ 180 Days

Variable (units)	<180 Days (% of results above upper limit of normal n=211	≥180 Days (% of results above upper limit of normal n=288	Odds Ratio	95% CI	p-value
AST (U/L)	4.7% (8/172)	6.5% (17/262)	2.40	(1.00, 5.74)	0.05
Total Bilirubin (mg/dL)	7.9% (14/178)	15.1% (40/265)	2.46	(1.26, 4.81)	0.009
Lymphocytes Absolute (K/mcL)	3.8% (7/182)	5.6% (15/270)	2.90	(1.18, 7.13)	0.02
	**(% of results below lower limit of normal)**	**(% of results below lower limit of normal)**			
C4 (mg/dL)	11.2% (20/178)	3.8% (10/264)	0.33	(0.15, 0.72)	0.005

Percentage of infected participants having abnormal laboratory values, with odds ratios of abnormal results comparing infected participants whose baseline visit was less than 180 days from SARS-CoV-2 infection to infected participants whose baseline visit was 180 days or more. p-values comparing odds of the findings are adjusted for age and sex. Generalized estimating equations (GEE) are used to account for correlation between family members participating in the study.
Abbreviations: CI (confidence interval), U/L (units per liter), mg/dL (milligrams per deciliter), K/mcL (thousand cells per cubic millimeter.

eTable 16: Comparison SARS-CoV-2 PCR, Anti-nucleocapsid and Total Neutralizing Antibodies between Infected and Uninfected Participants

Variable	Uninfected No (%)	Infected No (%)	GEE Coef	95% CI	p-value
Positive SARS-CoV-2 PCR nasal swab	0.0% (0/111)	1.7% (9/524)	Inf	(0.42, Inf)	0.3
Positive SARS-CoV-2 PCR stool	0.0% (0/39)	1.9% (3/156)	Inf	(0.10, Inf)	>.99
Positive Anti-Nucleocapsid Antibody	0.0% (0/110)	80.5% (396/492)	Inf	(118.15, Inf)	<.001
Positive Total Neutralizing Antibody	78.2% (86/110)	87.9% (451/513)	2.40	(1.37, 4.20)	0.002

Results are shown in percentages for infected and uninfected participants. P-values are computed using linear models comparing mean results between infected and uninfected, adjusting for age and sex. Generalized estimating equations (GEE) are used to account for correlation between family members participating in the study. Abbreviations: CI (confidence interval), Coef (coefficient), PCR (polymerase chain reaction), N/A (Not applicable).

eFigure 7: Comparison of SARS-CoV-2 Total Neutralizing Antibody with Vaccination Status between Infected and Uninfected Participants

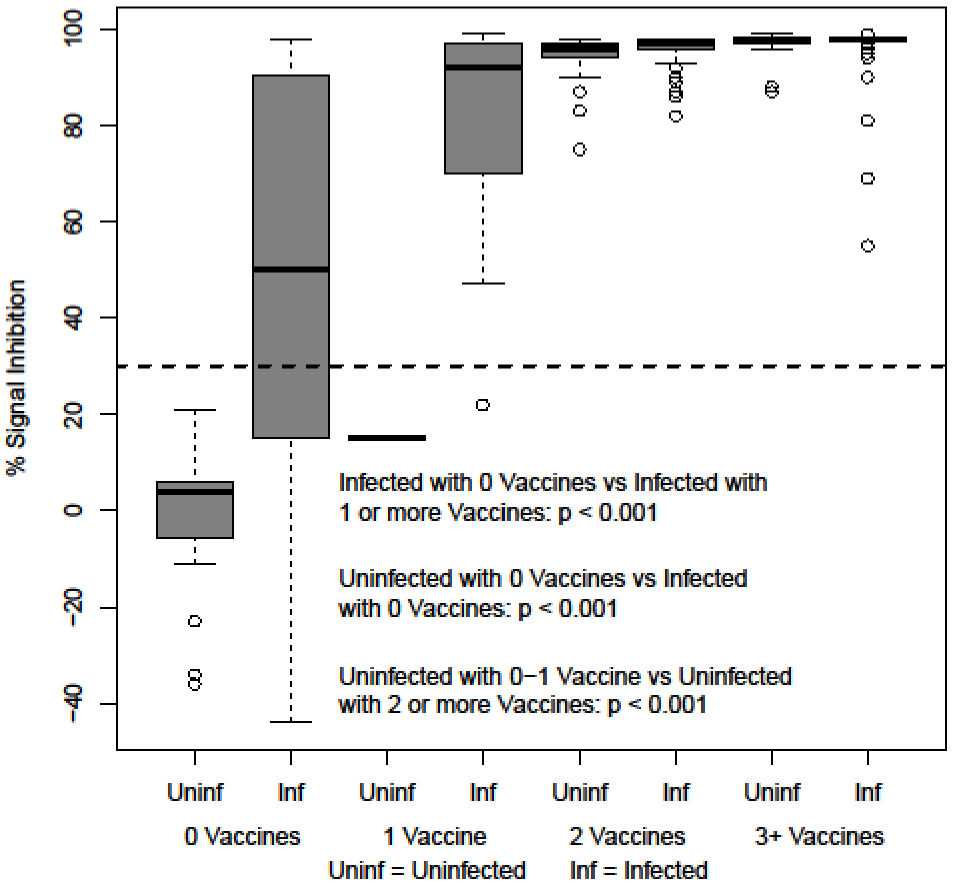

Genescript SARS-CoV-2 neutralizing antibody activity among infected and uninfected participants, prior to vaccination and after vaccination are depicted graphically. Dotted line represents 30% of SARS-CoV-2 inhibition, values equal or greater than 30% are consider positive. Shaded box indicates cohort IQR; horizontal bar within the box indicates median % signal inhibition (neutralizing antibody activity).

References

1 Fuchs, S. I., Eder, J., Ellemunter, H. & Gappa, M. Lung Clearance Index: Normal Values, Repeatability, and Reproducibility in Healthy Children and Adolescents. *Pediatr Pulmonol* **44**, 1180-1185 (2009).

2 Stanojevic, S. et al. Ers/Ats Technical Standard on Interpretive Strategies for Routine Lung Function Tests. *Eur Respir J* **60** (2022).

3 Holland, A. E. et al. An Official European Respiratory Society/American Thoracic Society Technical Standard: Field Walking Tests in Chronic Respiratory Disease. *Eur Respir J* **44**, 1428-1446 (2014).

4 Graham, B. L. et al. 2017 Ers/Ats Standards for Single-Breath Carbon Monoxide Uptake in the Lung. *Eur Respir J* **49** (2017).

5 Lopez, L. et al. Recommendations for Quantification Methods During the Performance of a Pediatric Echocardiogram: A Report from the Pediatric Measurements Writing Group of the American Society of Echocardiography Pediatric and Congenital Heart Disease Council. *J Am Soc Echocardiogr* **23**, 465-495; quiz 576-467 (2010).

6 Lang, R. M. et al. Recommendations for Chamber Quantification: A Report from the American Society of Echocardiography's Guidelines and Standards Committee and the Chamber Quantification Writing Group, Developed in Conjunction with the European Association of Echocardiography, a Branch of the European Society of Cardiology. *J Am Soc Echocardiogr* **18**, 1440-1463 (2005).

7 McCrindle, B. W. et al. Diagnosis, Treatment, and Long-Term Management of Kawasaki Disease: A Scientific Statement for Health Professionals from the American Heart Association. *Circulation* **135**, e927-e999 (2017).

8 Harahsheh, A. S. et al. Cardiac Echocardiogram Findings of Severe Acute Respiratory Syndrome Coronavirus-2-Associated Multi-System Inflammatory Syndrome in Children. *Cardiol Young* **32**, 718-726 (2022).

9 Bevans, M., Ross, A. & Cella, D. Patient-Reported Outcomes Measurement Information System (Promis): Efficient, Standardized Tools to Measure Self-Reported Health and Quality of Life. *Nurs Outlook* **62**, 339-345 (2014).

10 Nikolaidis, A. et al. The Coronavirus Health and Impact Survey (Crisis) Reveals Reproducible Correlates of Pandemic-Related Mood States across the Atlantic. *Sci Rep* **11**, 8139 (2021).
